# Brain‐Wide Spatiotemporally Distinct Traveling Waves Drive Anxiety‐Like Behaviors in Mice

**DOI:** 10.1002/advs.202411867

**Published:** 2025-07-29

**Authors:** Jiaming Liu, Jia‐Wen Mo, Xunda Wang, Yinuo Ma, Shile Tian, Qi Wang, Peng‐Li Kong, Ziqi An, Li Ding, Jing Ren, Cheng‐Lin Lu, Chuanjun Tong, Ed X. Wu, Qiu‐gen Hu, Xiong Cao, Yanqiu Feng

**Affiliations:** ^1^ Key Laboratory of Mental Health of the Ministry of Education Guangdong‐Hong Kong‐Macao Greater Bay Area Center for Brain Science and Brain‐Inspired Intelligence Guangdong‐Hong Kong Joint Laboratory for Psychiatric Disorders Guangdong Province Key Laboratory of Psychiatric Disorders Guangdong Basic Research Center of Excellence for Integrated Traditional and Western Medicine for Qingzhi Diseases Department of Neurobiology School of Basic Medical Sciences Southern Medical University Guangzhou 510515 China; ^2^ Department of Radiology The Eighth Affiliated Hospital Southern Medical University (The First People's Hospital of Shunde) Foshan 528300 China; ^3^ Guangxi Key Laboratory of Brain and Cognitive Neuroscience Faculty of Basic Medical Sciences Guilin Medical University Guilin Guangxi 541199 China; ^4^ Department of Neurosurgery Stanford University School of Medicine Stanford CA 94304 USA; ^5^ School of Biomedical Engineering Guangdong Provincial Key Laboratory of Medical Image Processing Southern Medical University Guangzhou 510515 China; ^6^ Microbiome Medicine Center Department of Laboratory Medicine Zhujiang Hospital Southern Medical University Guangzhou Guangdong 510515 China; ^7^ Institute of Neuroscience CAS Key Laboratory of Primate Neurobiology Center for Excellence in Brain Science and Intelligence Technology Chinese Academy of Sciences Shanghai 200031 China; ^8^ Laboratory of Biomedical Imaging and Signal Processing Department of Electrical and Electronic Engineering The University of Hong Kong Pokfulam Hong Kong SAR China

**Keywords:** anxiety, cortical spreading depression, cortical traveling waves, optogenetic fMRI

## Abstract

Cortical traveling waves coordinate communication among distributed neural ensembles to modulate brain function and dysfunction through distinct spatiotemporal propagation patterns. However, the brain‐wide propagation dynamics of traveling waves from different origins and their roles in regulating behavior remain unclear. Using optogenetics alongside whole‐brain fMRI in mice, it is demonstrated that optogenetic activation of the medial prefrontal cortex and primary somatosensory area induces cortical spreading depression (CSD)‐like traveling waves. These waves propagate beyond the ipsilateral cortex to the contralateral cortex and midbrain, reaching subcortical structures along cortico‐amygdala‐striatal pathways, ultimately terminating in the striatum ≈4 min after induction. The propagation directions in the cortex, speed, duration, and region involvement vary with wave origins. Furthermore, these CSD‐like traveling waves induce anxiety‐like behaviors and increase dendritic spine density. The findings elucidate the complete process from induction to termination of traveling waves across the whole brain and reveal their previously undiscovered role in driving anxiety.

## Graphical abstract

J.Liu et al. reveal the complete propagation dynamics of cortical traveling waves, from local circuitry initiation to long‐range propagation, and identify their functional relevance in modulating anxiety‐like behaviors and underlying cellular neuroplasticity in mice.

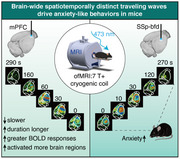

## 1 Introduction

Spatiotemporal coordination of neural activity across distributed brain regions is crucial for brain function. Traveling waves have been proposed to regulate communication among neuronal ensembles in anatomically distributed brain regions.^[^ ^1^, ^2^ ^]^ These waves are prevalent, having been observed in various species (including turtles, birds, rodents, monkeys, and humans),^[^ ^3^, ^4^, ^5^, ^6^, ^7^, ^8^ ^]^ across different brain sites,^[^ ^3^, ^9^, ^10^, ^11^ ^]^ and in diverse brain states (awake, anesthetized, and hallucinating).^[^ ^12^, ^13^ ^]^ Traveling waves propagate across a broad range of spatial and temporal scales. As manifestations of propagating neuronal activity, their spatiotemporal organization reveals the sequence of brain region activation and the duration of dynamic neuronal coordination, ultimately influencing brain function and behavioral outcomes.^[^ ^2^ ^]^ For example, their properties such as propagation pathways, direction, duration, and frequency bands, have been linked to reward history encoding,^[^ ^5^ ^]^ memory processes and consolidation,^[^ ^3^, ^14^ ^]^ visual perception,^[^ ^7^, ^9^, ^15^, ^16^ ^]^ attention,^[^ ^17^ ^]^ cognition,^[^ ^18^ ^]^ and motor behavior.^[^ ^19^ ^]^ Moreover, alterations in the propagation properties of traveling waves have been clinically associated with disorders such as memory deficits,^[^ ^20^ ^]^ schizophrenia,^[^ ^21^ ^]^ and attention‐deficit/hyperactivity disorder.^[^ ^22^ ^]^ Thus, interrogating the spatiotemporal dynamics of traveling waves is essential for understanding their role in modulating behavioral performance in both health and disease.

Previous attempts to investigate the spatiotemporal properties of traveling waves have predominantly employed electrophysiological and wide‐field optical imaging approaches. These studies have identified several properties of traveling waves, such as direction and frequency bands, in locally isolated brain regions, including the somatosensory cortex,^[^ ^6^, ^11^, ^23^ ^]^ primary visual cortex,^[^ ^15^, ^24^, ^25^, ^26^, ^27^ ^]^ hippocampus,^[^ ^28^, ^29^, ^30^ ^]^ and others.^[^ ^10^, ^31^, ^32^ ^]^ However, many spontaneously propagating traveling waves extend from their sources to distal areas, covering spatial scales from a single cortical region to multiple structures.^[^ ^2^ ^]^ Additionally, the propagation direction of traveling waves is largely unconstrained by underlying axonal projections.^[^ ^2^, ^25^, ^33^ ^]^ Despite known anatomical connectivity between brain regions, these factors make it an insurmountable task to determine or infer spatiotemporal properties of traveling waves beyond the recorded brain regions, such as long‐range propagation pathways. Therefore, it is difficult to elucidate key properties of traveling waves, such as their duration and propagation pathways throughout the brain, particularly in deep subcortical structures.

Furthermore, previous studies suggested that the propagation properties of traveling waves may be related to their origins.^[^ ^34^ ^]^ Previous studies for mapping traveling wave propagation are passive observations and cannot identify the origin of traveling waves.^[^ ^34^, ^35^, ^36^ ^]^ Consequently, detected waves are often a mixture of multiple complex waves with unknown origins,^[^ ^36^, ^37^ ^]^ complicating the analysis of the spatiotemporal propagation dynamics of traveling waves. However, the brain‐wide spatiotemporal propagation properties of individual traveling waves with known origins and their potential functional significance remain unexplored.

Cortical spreading depression (CSD) is a typical traveling wave frequently associated with numerous serious brain disorders in humans, including migraine with aura,^[^ ^38^, ^39^, ^40^, ^41^ ^]^ stroke,^[^ ^39^, ^42^, ^43^ ^]^ intracerebral hemorrhage,^[^ ^44^ ^]^ traumatic brain injury,^[^ ^44^ ^]^ and epilepsy.^[^ ^45^ ^]^ CSD exhibits typical propagation properties of traveling waves, such as spontaneous propagation largely unconstrained by axonal projection.^[^ ^46^, ^47^ ^]^ Meanwhile, CSD propagates slowly and causes robust brain activation, overcoming the limitations of temporal resolution and sensitivity of imaging techniques. These intrinsic propagation properties make CSD well‐suited for studying the propagation dynamics of individual traveling waves on a whole‐brain scale.

In this study, we used CSD as a model to investigate the spatiotemporal propagation properties of traveling waves induced from different origins across the whole brain, and their effects on behavioral performance in mice. Previous studies have shown that the sensory cortex is highly susceptible to CSD induction in both humans and mice, especially the whisker barrel region in mice.^[^ ^48^, ^49^ ^]^ As a result, the sensory cortex serves as one of the most frequently studied brain areas in CSD research.^[^ ^50^ ^]^ In addition, imaging studies have reported structural and functional abnormalities in the prefrontal cortex in patients with CSD‐related disorders.^[^ ^51^ ^]^ Therefore, we first tested whether optogenetic activation of excitatory neurons in the primary somatosensory area, barrel field (SSp‐bfd), and medial prefrontal cortex (mPFC) in mice could induce CSD‐like traveling waves. We then examined and compared the propagation, termination, speed, duration, and brain region involvement of mPFC‐ and SSp‐bfd‐induced traveling waves on a whole‐brain scale. Finally, we evaluated the impact of traveling wave propagation on neuronal plasticity and its association with behavioral performance.

## 2 Result

### 2.1 mPFC‐Induced Traveling Waves Exhibit Anterior‐to‐Posterior Cortico‐Cortical Propagation, with Parallel Interhemispheric Spread and Subsequent Cortico‐Subcortical Propagation

**Figure 1:**
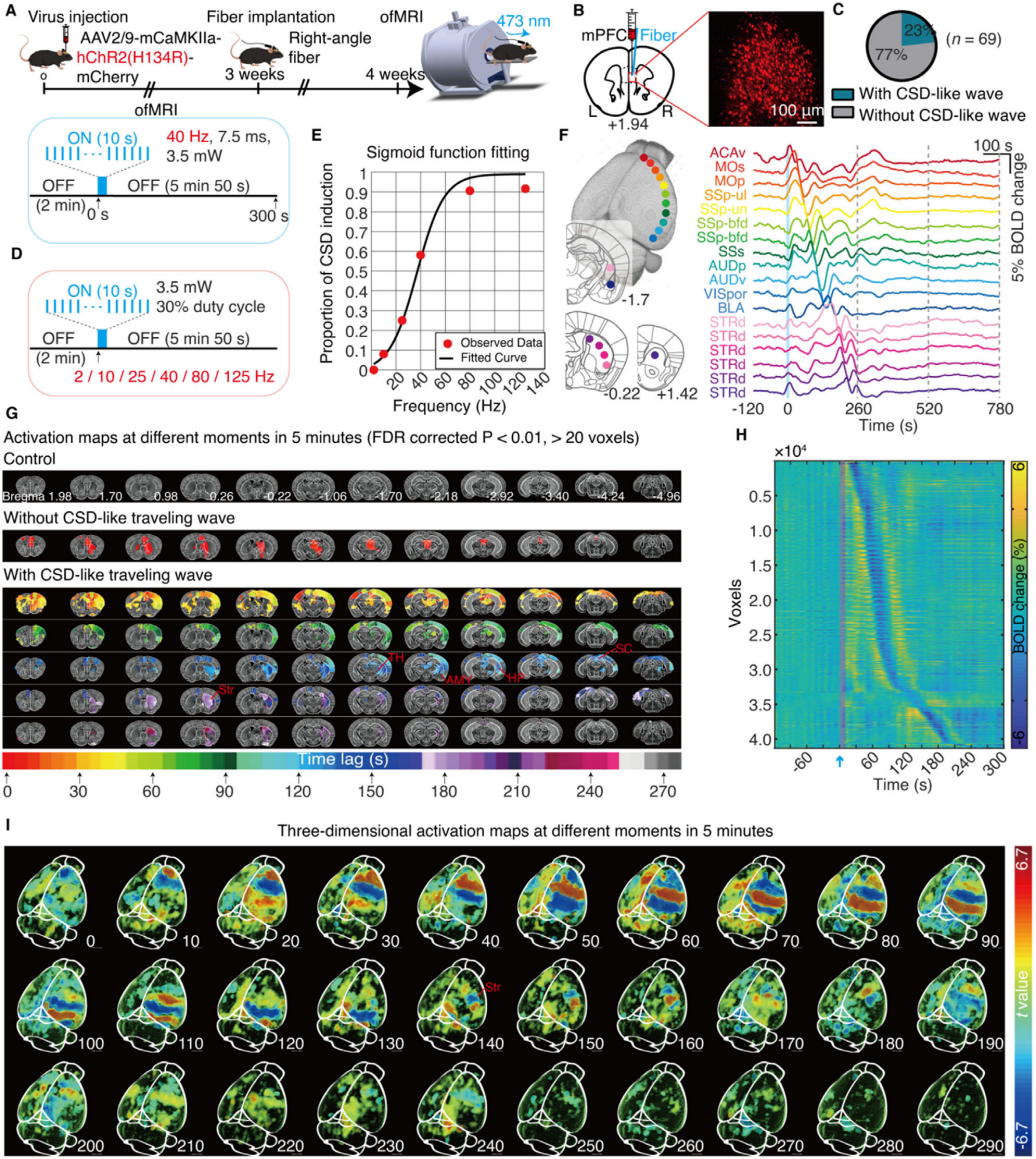
Optogenetic activation of the medial prefrontal cortex (mPFC) induces cortical traveling waves. A) Top: Schematic illustration depicting the timeline of unilateral viral injection, optical fiber implantation, and fMRI experiments. Bottom: Diagram of the ofMRI scanning paradigms. B) Confocal images showing ChR2 expression in mPFC neurons. C) Percentage of CSD‐like traveling waves induced by 40 Hz optogenetic activation of the mPFC. D) Six different light frequencies were randomly delivered to the mPFC at 3.5 mW light power with a 30% duty cycle. E) Logistic function fitting shows the stimulation frequency‐dependent induction probability of traveling waves (*R^2^* = 0.9828; slope = 0.091; threshold frequency = 37 Hz). F) Mean BOLD signals of 18 regions of interest (ROIs) in the ipsilateral brain of a representative mouse. See Table S1 (Supporting Information) for details on abbreviations. G) Activation maps at various time points during and after optogenetic stimulation of the mPFC in mCherry‐control mice (*n* = 9), representative mice without and with mPFC‐induced traveling waves. H) Voxel‐wise BOLD signals of mPFC‐induced traveling waves from a representative mouse, sequentially displayed in order of the time delay corresponding to the maximum correlation coefficient in the cross‐correlation analysis between the hemodynamic response function and the time series. I) 3D maximum intensity projection images from a representative mouse, showing the propagation pathway of mPFC‐induced traveling waves. Abbreviations: Str, striatum; AMY, amygdala; HP, hippocampus; TH, thalamus; SC, superior colliculus.

To induce traveling waves with in vivo optogenetic activation of mPFC, we unilaterally injected AAV‐CamkIIα‐hChR2(H134R)‐mCherry or a control virus (AAV‐CamkIIα‐mCherry) into the mPFC of naïve adult male C57BL/6J mice. After 3 weeks of viral expression, we implanted an optical fiber ≈0.1 mm above the infected neurons for light delivery (**Figure** **1**A). Confocal imaging confirmed ChR2 expression in mPFC neurons (Figure 1B). Previous studies have shown that 40 Hz gamma rhythmic sensory or optogenetic stimulation can induce network oscillations and traveling waves that coordinate neural activity at the network level.^[^ ^52^, ^53^, ^54^, ^55^, ^56^, ^57^ ^]^ Thus, we hypothesized that 40 Hz stimulation is a critical frequency for inducing traveling waves. Combining optogenetics and functional magnetic resonance imaging (ofMRI), we activated ChR2‐expressing neurons in the mPFC with 40 Hz optogenetics while simultaneously monitoring whole‐brain neural activity. We demonstrated that 40 Hz optogenetic activation (473 nm, 10 s, 3.5 mW, 30% duty cycle) of the mPFC induced traveling waves in 23% of ChR2‐expressing mice (16 out of 69 mice; Figure 1C). These traveling waves can be directly detected through visual inspection of the raw series images (Video S1, Supporting Information), which is characterized by sequential activation of brain regions and appears as a cortical wave of BOLD signal changes propagating across different regions over time. We also checked the presence of traveling waves by calculating brain activation maps. No traveling waves were observed in mCherry‐control mice (*n* = 9 mice; Figure 1G).

In a separate experiment, we sought to verify the effect of optogenetic stimulation frequency on the probability of traveling wave induction. We randomly delivered six optogenetic stimulation frequencies (2, 10, 25, 40, 80, or 125 Hz) in mice that had shown successful traveling waves induction through 40‐Hz optogenetic stimulation in a prior ofMRI experiment targeting the mPFC (*n* = 16 mice; Figure 1D). Only one of these frequencies was delivered per trial, and each mouse received 9 to 10 trials. The proportion of trials that successfully induced traveling waves reached ≈91% at 80 Hz and 92% at 125 Hz, respectively. Furthermore, optogenetic stimulation frequencies as low as 10 Hz and 25 Hz also induced traveling waves, although at lower proportions: 8% at 10 Hz and 25% at 25 Hz. No traveling waves were induced with 2 Hz light stimulation of the mPFC (Figure 1E). We used a Logistic function to model the relationship between optogenetic frequency and the traveling wave induction probability. The coefficient of determination (*R* ^2^ = 0.9828) is close to 1, indicating that the logistic model accurately describes the data trend. The fitted threshold frequency was 37 Hz, close to 40 Hz. These results suggest that optogenetic stimulation frequency influences the probability of traveling wave induction, and 40 Hz frequency appears to be a key threshold frequency for effectively inducing traveling waves (Figure 1E). Notably, mice that were successfully induced to travel by 40 Hz optogenetic activation of the mPFC were able to induce traveling waves again one week later using the same stimulation parameters (*n* = 7 mice; Figure S1, Supporting Information), indicating high reproducibility of optogenetically induced traveling waves.

We quantified functional magnetic resonance imaging (fMRI) responses using the beta values from general linear model (GLM) analysis for both mice with and without traveling waves at 40 Hz stimulation. The brain was divided into 68 regions of interest (ROIs) covering 11 networks, based on the Allen Mouse Brain Atlas (Figure S2A, Supporting Information). Using the brain‐wide monosynaptic axonal projections data of the mPFC from the Allen Mouse Brain Connectivity Atlas (http://connectivity.brain‐map.org/),^[^ ^58^ ^]^ we gained the ipsilateral structural connectivity of the mPFC (Figure S2D, Supporting Information). As expected, in mice without traveling waves, the brain regions activated by optogenetic stimulation were highly consistent with the main axonal projections of mPFC, mainly located in the prefrontal cortex, striatum, motor cortex, and brain stem (Figure S2E, Supporting Information). Notably, in mice without traveling waves, fMRI response strengths exhibited significant positive correlations with structural connectivity (*r* = 0.6378, *p* < 0.0001; Figure S2F, Supporting Information), whereas in mice with traveling waves, such correlations were not significant (*r* = 0.0115, *p* = 0.9271; Figure S2H, Supporting Information). These findings support prior conclusions that the involvement of brain regions in traveling waves is largely not determined by axonal projections.^[^ ^2^, ^25^, ^33^ ^]^

The mPFC‐induced traveling waves consistently propagated from the cortex to the cerebellum in the ipsilateral hemisphere along the anterior‐to‐posterior direction for ≈140 s. Traveling waves also involved the contralateral cortex, ipsilateral subcortical structures, and the midbrain regions, particularly the hippocampus, amygdala, thalamus, superior colliculus, and striatum (Figure 1G,I; Figure S3, Supporting Information). These waves spread along a posterior‐to‐anterior direction through the striatum for ≈90 s before terminating in the anterior striatum (Figure 1G,I; Figure S3, Supporting Information). The total propagation duration exceeded 4 min (Figure 1G–I). A 3D dynamic video in Video S2 (Supporting Information) displays the complete process of induction, propagation, to termination of individual mPFC‐induced traveling waves throughout the brain. These results demonstrate that our ofMRI approach enables the localized induction of traveling waves while simultaneously capturing their complete propagation dynamics across the whole brain from a known origin.

### 2.2 SSp‐bfd‐Induced Traveling Waves Exhibit Parallel Multidirectional Cortico‐Cortical Propagations Followed by Cortico‐Subcortical Propagations

**Figure 2:**
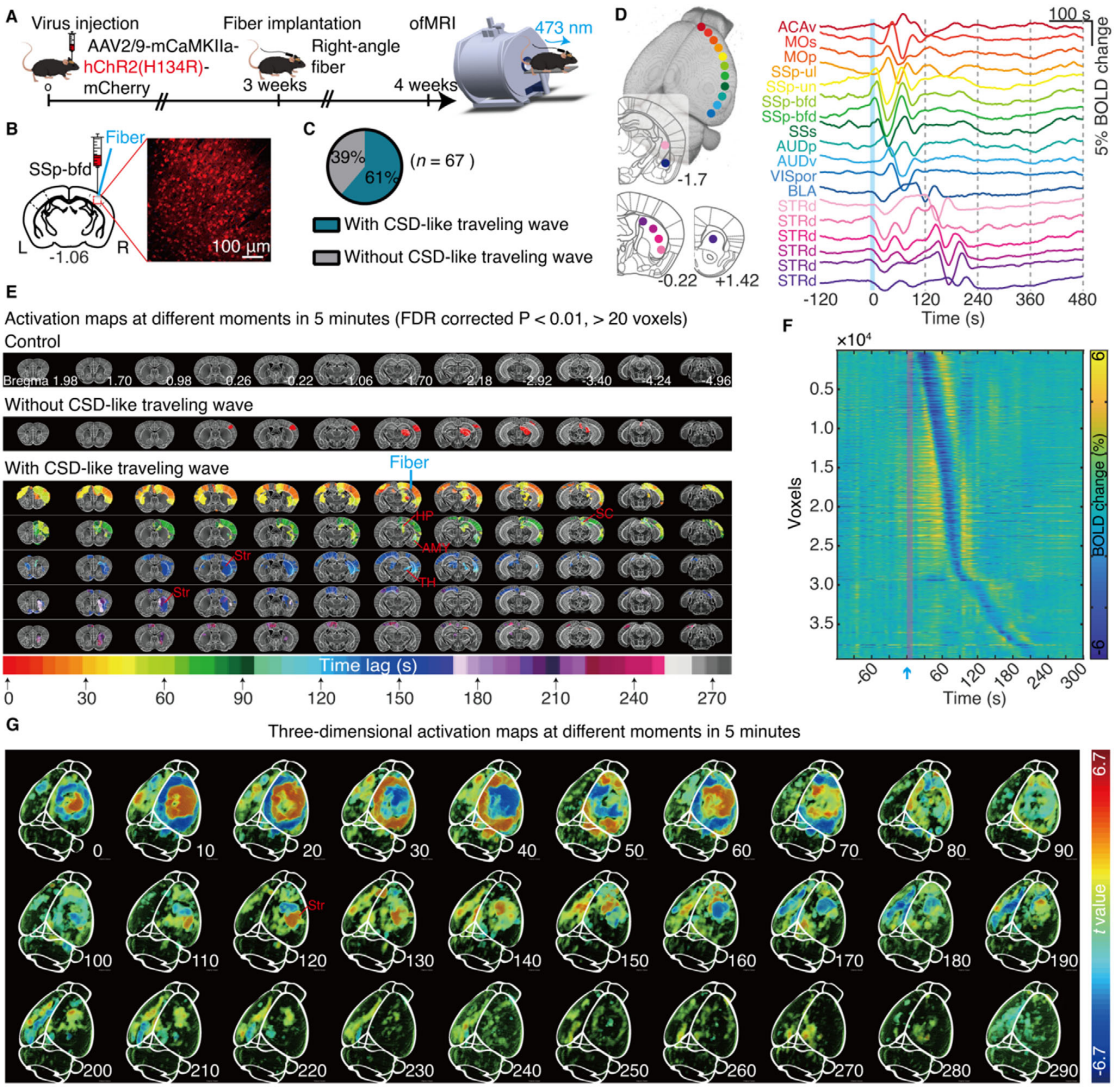
Optogenetic activation of the primary somatosensory area, barrel field (SSp‐bfd), induces cortical traveling waves. A) Schematic timeline illustrating unilateral viral injection, optical fiber implantation, and ofMRI experiments. B) Confocal images showing ChR2 expression in SSp‐bfd neurons. C) Pie chart depicting the percentage of the CSD‐like traveling waves induced by 40 Hz optogenetic activation of SSp‐bfd. D) Mean BOLD signals of 18 ROIs in the ipsilateral brain of a representative mouse. For details on abbreviations, see Table S1 (Supporting Information). E) Activation maps at different time points during and after optogenetic activation of SSp‐bfd in mCherry controls (*n* = 10 mice), representative mice without and with SSp‐bfd‐induced traveling waves. F) Voxel‐wise BOLD signals of SSp‐bfd‐induced traveling waves from representative mice, sequentially displayed in order of the time delay corresponding to the maximum correlation coefficient in the cross‐correlation analysis between the hemodynamic response function and the time series. G) 3D maximum intensity projection images of a representative mouse illustrating the propagation pathway of SSp‐bfd‐induced traveling waves. Abbreviations: Str, striatum; AMY, amygdala; HP, hippocampus; TH, thalamus; SC, superior colliculus.

Next, we tested whether the propagation properties of traveling waves depend on their origins, i.e., whether traveling waves induced in different brain regions exhibit distinct propagation patterns. To investigate this, we unilaterally injected AAV‐CamkIIα‐hChR2(H134R)‐mCherry or AAV‐CamkIIα‐mCherry into the SSp‐bfd in naïve adult male C57BL/6J mice and repeated the ofMRI experiments conducted in the mPFC (**Figure** **2**A). Confocal imaging confirmed ChR2 expression in SSp‐bfd neurons (Figure 2B). We found that optogenetic activation of the SSp‐bfd induced traveling waves in 61% of ChR2‐expressing mice with 40 Hz light stimulation (473 nm, 10 s, 3.5 mW, 30% duty cycle) (Figure 2C), indicating a higher induction probability compared to the mPFC (23%, Figure 1C). The induction probability in female mice was 59%, which was comparable to that in male mice (61%) (Figure S4B, Supporting Information). No traveling waves were induced in mCherry‐control mice under the same stimulation parameters (Figure 2E).

SSp‐bfd‐induced traveling waves diffusely spread from the stimulation site along the surrounding cortex in all directions within the ipsilateral hemisphere for ≈100 s (Figure 2G), unlike mPFC‐induced traveling waves, which propagated in an anterior‐to‐posterior direction across the cortex. SSp‐bfd‐induced traveling waves also spread to the contralateral cortex, midbrain regions, and subcortical structures, including the hippocampus, amygdala, thalamus, superior colliculus, and striatum (Figure 2E,G; Figure S5, Supporting Information). SSp‐bfd‐induced traveling waves spread to the striatum after ≈100 s, where they propagate in a posterior‐to‐anterior direction for ≈100 s before terminating in the anterior striatum (Figure 2G; Figure S5, Supporting Information). The entire propagation of SSp‐bfd‐induced traveling waves lasted less than 4 min (Figure 2E–G), shorter than the propagation of mPFC‐induced traveling waves. Video S3 (Supporting Information) illustrates the complete brain‐wide dynamic propagations of individual SSp‐bfd‐induced traveling waves. These results demonstrate that while both mPFC‐ and SSp‐bfd‐induced traveling waves involve multiple brain regions, including the bilateral cortex, midbrain, and subcortical structures, they exhibit distinct propagation patterns across the cortex and differ in propagation duration.

### 2.3 Origin‐ and Induction‐Order‐Dependent Traveling Wave Propagation Speed and Brain Region Involvement

**Figure 3:**
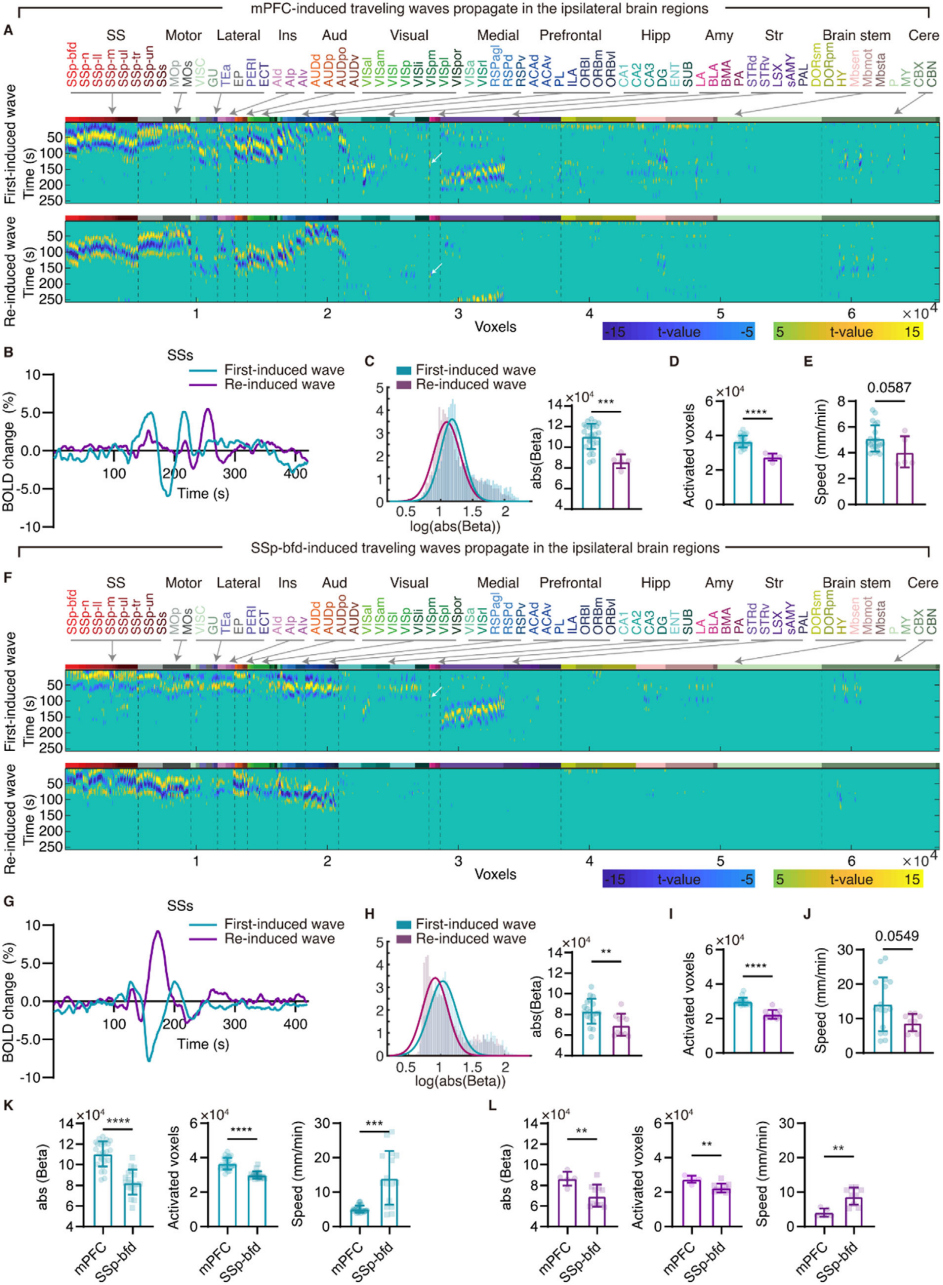
Comparison of ipsilateral propagation properties between mPFC‐ and SSp‐bfd‐induced traveling waves. A) Voxel‐wise spatiotemporal activation of mPFC first‐ (Top, a representative mouse) and re‐induced traveling waves (bottom, a representative mouse). B) The BOLD signals in the secondary somatosensory cortex (SSs) of two representative mice undergoing mPFC first‐ and re‐induced traveling wave propagation, respectively. C–E) Quantitative comparisons of the brain responses (C), activated voxels (D), and propagation speed in the cortex (E) between mPFC first‐ (*n* = 26) and re‐induced (*n* = 5) traveling waves. F) Voxel‐wise spatiotemporal activation of SSp‐bfd first‐ (Top, a representative mouse) and re‐induced traveling waves (bottom, a representative mouse). G) The BOLD signals in the SSs of two representative mice undergoing SSp‐bfd first‐ and re‐induced traveling wave propagation, respectively. H–J) Quantitative comparisons of the brain responses (H), activated voxels (I), and propagation speed in the cortex (J) between SSp‐bfd first‐ (*n* = 19) and re‐ (*n* = 10) induced traveling waves. K–L) Quantitative comparisons of the brain responses, activated voxels, and propagation speed in the cortex between mPFC‐ and SSp‐bfd‐induced traveling waves. See Table S1 (Supporting Information) for the details of other abbreviations. White arrows emphasize that the lateral amygdala (LA) is activated first before the dorsal striatum (STRd) is activated. The Mann‐Whitney test was used for (J) and the speed in (K), while two‐sample t‐tests were applied to other comparisons. ^**^ *p* < 0.01, ^***^ *p* < 0.001; ^****^ *p* < 0.0001. Data are presented as mean ± std.

Next, we evaluated the fMRI responses, propagation speed, and brain region involvement of traveling waves, and compared these properties across traveling waves induced in different origins. Previous studies have indicated that first‐ and re‐induced CSD‐like traveling waves exhibit distinct effects on CSD‐related disorders,^[^ ^59^, ^60^, ^61^ ^]^ suggesting that traveling waves might exhibit induction order‐dependent properties. To test this hypothesis first, we quantitatively compared the propagation properties between first‐ and re‐induced CSD‐like traveling waves within a single ofMRI experiment. We found that the brain showed different activation patterns for first‐ and re‐induced CSD‐like traveling waves over the time course (**Figure** **3**A,F; Figure S6 and S7, Supporting Information). In first‐induced CSD‐like traveling waves, ipsilateral brain regions showed deactivation, activation, and deactivation responses, whereas in re‐induced CSD‐like traveling waves, these brain regions were first activated, deactivated, and then activated again. These results support a previous study showing that first‐ and re‐induced CSD‐like traveling waves trigger different brain perfusion profiles.^[^ ^62^ ^]^

We found that both mPFC‐ and SSp‐bfd‐induced first CSD‐like traveling waves produced significantly greater BOLD responses (ipsilateral: mPFC, *p* = 0.0002; SSp‐bfd, *p* = 0.008, Figure 3C,H; contralateral: mPFC, *p* = 0.0221; SSp‐bfd, *p* = 0.0009, Figure S8C,D, Supporting Information) and higher BOLD signal amplitudes (mPFC, *p* = 0.0005; SSp‐bfd, *p* < 0.0001; Figure S9B,C, Supporting Information) compared to re‐induced CSD‐like traveling waves in both the ipsilateral and contralateral cortex. Furthermore, first‐induced CSD‐like traveling waves activated significantly more brain regions than re‐induced waves in both cerebral hemispheres (ipsilateral: mPFC, *p* < 0.0001; SSp‐bfd, *p* < 0.0001, Figure 3D,I; contralateral: mPFC, *p* = 0.0029; SSp‐bfd, *p* = 0.0003, Figure S8C,D, Supporting Information). Notably, compared to first‐induced CSD‐like traveling waves, re‐induced waves induced by optogenetic activation of the SSp‐bfd did not propagate to subcortical structures (e.g., the hippocampus, amygdala, and striatum) or the brain stem (Figure 3F). Moreover, re‐induced CSD‐like traveling waves took significantly longer to propagate to the same ROIs (Figure 3A,F; mPFC, *p* = 0.0005; SSp‐bfd, *p* < 0.0001, Figure S10B,C, Supporting Information), showed a trend toward slower propagation speeds in the cortex (mPFC, *p* = 0.0587; SSp‐bfd, *p* = 0.0549, Figure 3E,J), and had an overall longer traveling duration. These results demonstrate that the propagation properties of traveling waves depend on the induction order (i.e., first or repeated induction).

We found that both first‐ and re‐induced mPFC‐induced traveling waves elicited significantly greater BOLD responses and activated significantly more brain regions compared to SSp‐bfd‐induced traveling waves both in ipsilateral (BOLD responses: First‐induced wave, *p* < 0.0001, Re‐induced wave, *p* = 0.0088; activated voxels: First‐induced wave, *p* < 0.0001, Re‐induced wave, *p* = 0.0019; Figure 3K,L) and contralateral cortex (BOLD responses: First‐induced wave, *p* < 0.0001, Re‐induced wave, *p* = 0.0016; activated voxels: First‐induced wave, *p* < 0.0001, Re‐induced wave, *p* = 0.0087; Figure S8E,F, Supporting Information). Notably, mPFC‐induced traveling waves, in contrast to those originating from the SSp‐bfd, activated more brain regions predominantly within the hippocampus, including CA1, CA2, and CA3 (Figure 3A,F), suggesting that traveling waves establish a specific pattern of neural communication between the prefrontal cortex and hippocampus. Furthermore, mPFC‐induced traveling waves propagated significantly slower in the cortex than SSp‐bfd‐induced traveling waves (First‐induced wave, *p* = 0.0002, Re‐induced wave, *p* = 0.0018, Figure 3K,L). Collectively, these results demonstrate that the key properties of traveling waves—such as the degree of brain activation, propagation speed, and brain region involvement—are dependent on both their origins and induction order.

### 2.4 Common Cortico‐Amygdala‐Striatal Termination Pathway for mPFC‐ and SSp‐bfd‐Induced Traveling Waves

**Figure 4:**
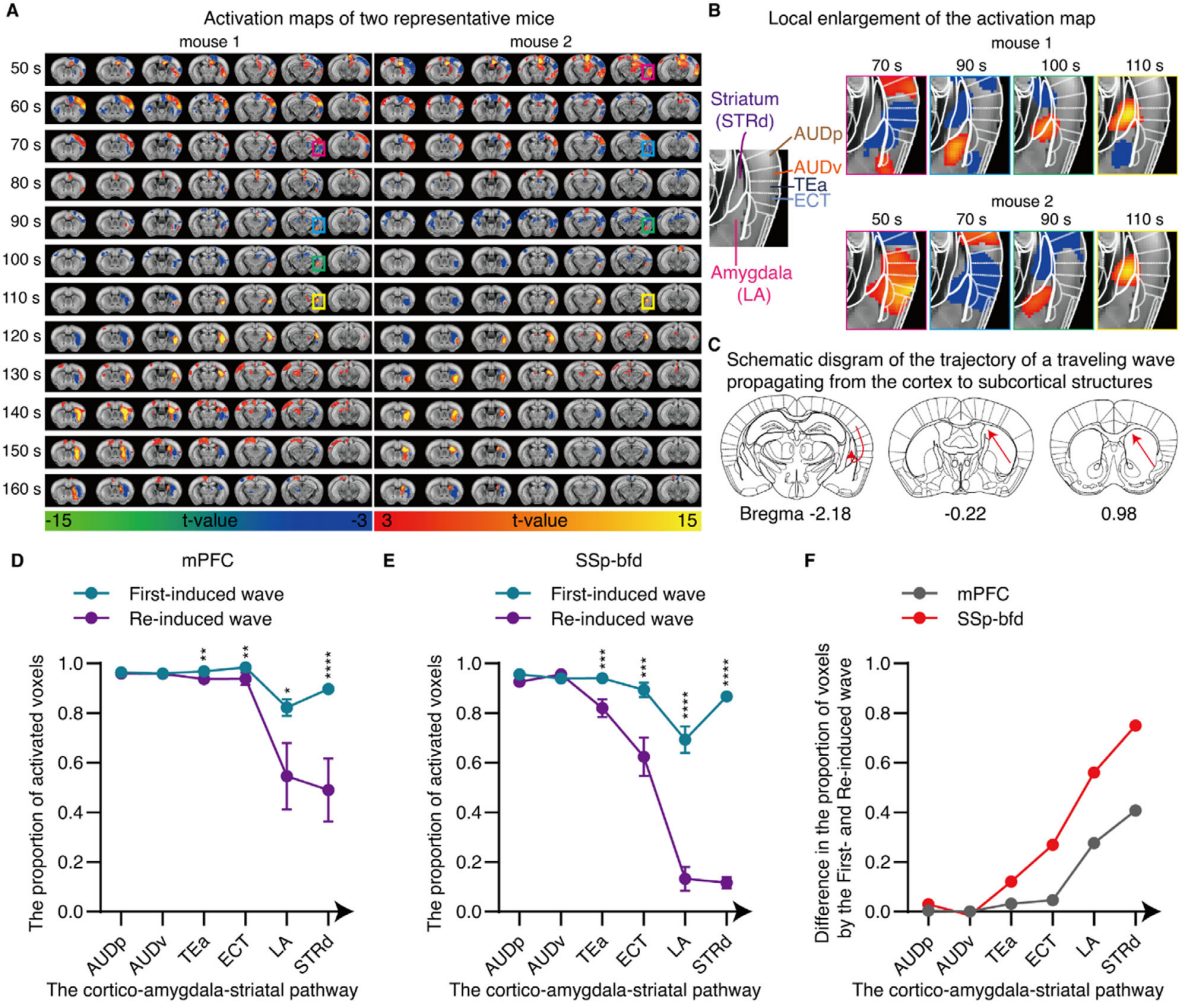
Cortical traveling waves propagate to the striatum through the amygdala. A,B) Activation maps A) and corresponding magnified views B) of two representative mice between 50 and 160 s after optogenetic activation of the SSp‐bfd. Abbreviations: AUDp, primary auditory area; AUDv, ventral auditory area; TEa, temporal association areas; ECT, ectorhinal area. C) Diagram showing the trajectory of traveling wave propagation. D‐E) Quantitative comparison of the proportion of voxels activated by first‐ and re‐induced waves in brain regions along the cortico‐amygdala‐striatal pathway. F) Difference in the proportion of voxels activated by first‐ and re‐induced traveling waves along the cortico‐amygdala‐striatal pathway. Mann–Whitney test. ^**^ *p* < 0.01, ^***^ *p* < 0.001; ^****^ *p* <  0.0001. Data are presented as mean ± s.e.m.

Where cortical traveling waves terminate their propagation has long been an unanswered question. We observed that the striatum, specifically its dorsal region (STRd), was the final area activated by both mPFC‐ and SSp‐bfd‐induced traveling waves (Figure 3A,F; Figure S10C, Supporting Information). These results demonstrate that traveling waves terminate their propagation in the striatum. Notably, we observed that traveling waves are consistently propagated in a posterior‐to‐anterior, bottom‐to‐top direction within the striatum (**Figure** **4**A; Figures S3–S5, Supporting Information). Moreover, the lateral amygdala nucleus (LA) consistently activated before striatal activation, both at the individual level (Figures 3A,F and 4A,B) and the group level (Figure S6B,D, Supporting Information). Videos S2 and S3 (Supporting Information), along with the activation maps at different time points, show that both mPFC‐ and SSp‐bfd‐induced traveling waves communicate to the striatum via a fixed cortico‐amygdala‐striatal propagation pathway (Figure 4C). These results reveal a previously unknown mechanism by which traveling waves convey information from the cortex to the striatum through the amygdala, highlighting an undiscovered form of corticostriatal connectivity.^[^ ^63^ ^]^

Compared to the first‐induced waves, the proportion of activated voxels along the cortico‐amygdala‐striatal pathway significantly decreased during re‐induced waves and exhibited a stepwise decline (Figure 4D,E), indicating a progressive weakening of wave‐induced activation strength. This reduction was particularly pronounced for waves induced by SSp‐bfd: the difference in the proportion of activated voxels between the first‐ and re‐induced waves was approximately twice the difference observed in the mPFC (Figure 4F). In particular, the proportion of activated voxels in the amygdala, a key relay station for cortico‐to‐subcortical propagation, was only 13.19% in SSp‐bfd re‐induced waves, which was significantly smaller than that in first‐evoked waves (69.24%), as well as in mPFC‐re‐induced waves (54.52%). These results suggest that a significant progressive reduction in activation strength along the cortico‐amygdala‐striatal pathway may account for the failure of SSp‐bfd re‐induced waves to propagate to subcortical brain regions (Figure 3F).

### 2.5 Traveling Waves Increase Dendritic Spine Density and Drive Anxiety‐Like Behaviors in Mice

Clinically, functional abnormalities in the sensory cortex may be more closely associated with CSD‐related disorders than those in the mPFC.^[^ ^38^, ^51^, ^64^ ^]^ For example, CSD‐like traveling wave is thought to be the pathophysiologic mechanism for migraine aura, which are primarily sensory symptoms, including paraesthesia, numbness of face and/or upper extremity, and scintillating scotoma, all of which are associated with sensory cortex functions.^[^ ^38^ ^]^ Numerous studies have shown that migraine is a disorder of abnormal sensory processing in the brain,^[^ ^64^ ^]^ associated with hypersensitivity and hyperexcitability of the sensory cortex.^[^ ^65^ ^]^ Moreover, patients with migraine exhibited abnormal functional connectivity in the pathway of primary somatosensory.^[^ ^51^ ^]^ In stroke, CSD‐like traveling wave propagation in the sensory cortex is a hallmark of secondary injury.^[^ ^44^ ^]^ Considering the importance of sensory cortex‐induced CSD‐like traveling waves for understanding the mechanisms of clinical CSD‐related disorders, we next investigate the effects of SSp‐bfd‐induced CSD‐like traveling waves on neuronal communication as well as behavioral performances in mice.

**Figure 5:**
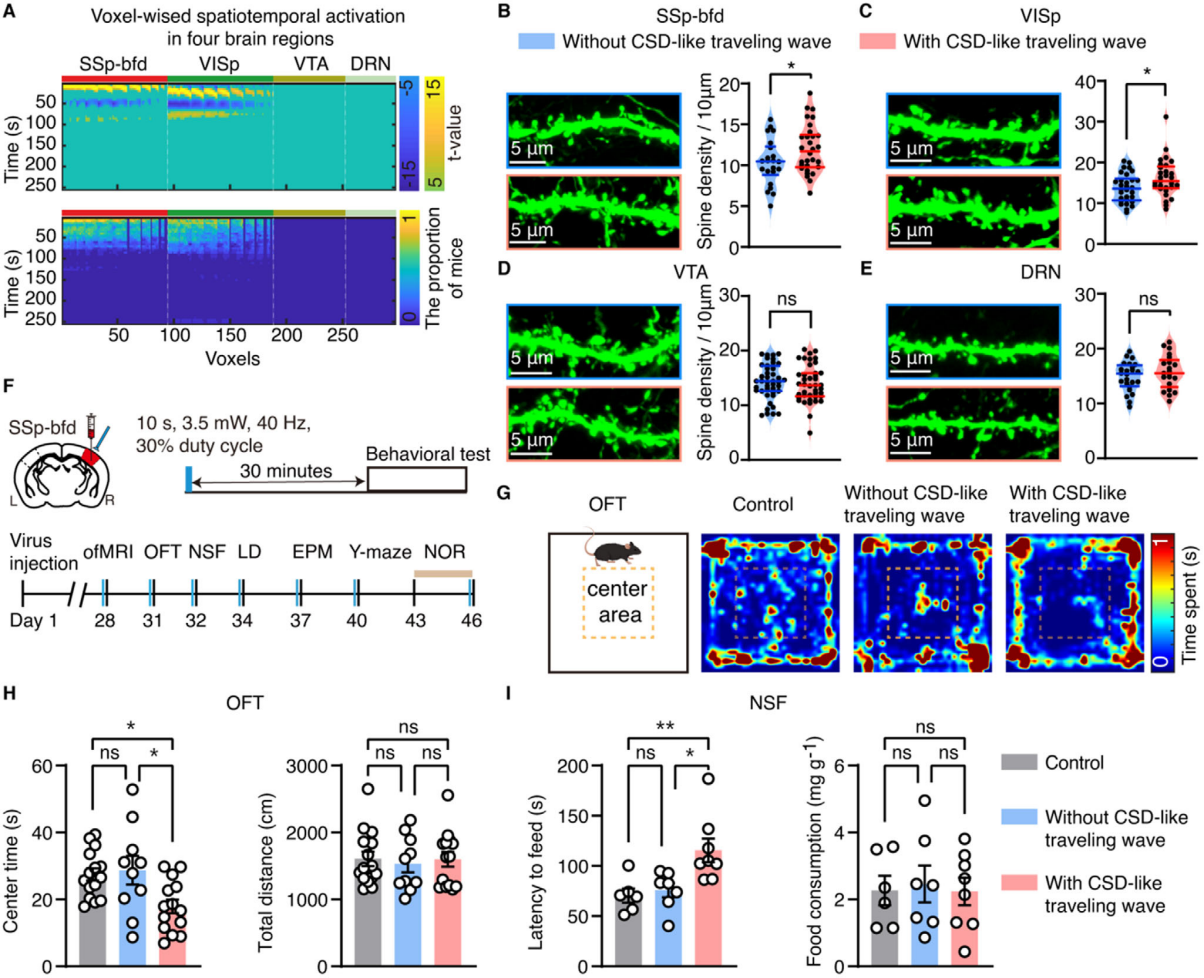
CSD‐like traveling waves increase dendritic spine density and drive anxiety‐like behaviors in mice. A) Top: voxel‐wise spatiotemporal activation map in four brain regions of a representative mouse, showing that SSp‐bfd‐induced CSD‐like traveling waves can propagate to ipsilateral SSp‐bfd and primary visual area (VISp), but not to the ventral tegmental area (VTA) and dorsal raphe nucleus (DRN). Bottom: The proportion of mice activated by SSp‐bfd‐induced CSD‐like traveling waves at different times in the ipsilateral four brain regions (*n* = 9 mice). B–E) Representative confocal images and quantitative analysis of dendritic spine density in Thy1‐YFP‐H transgenic mice with and without SSp‐bfd‐induced traveling waves. Two‐sample *t* tests. F) Schematic timeline of behavioral tests. G) Representative heatmaps of movement patterns of mice in the open field test (OFT). H,I) Quantitative analysis of behavioral performances in the OFT (H, *n* = 15, 10 and 14 for control, mice without CSD, and mice with CSD, respectively), and novelty‐suppressed feeding test (NSF, I; *n* = 6, 7 and 8 for control, mice without CSD, and mice with CSD, respectively). One‐way ANOVA followed by Tukey's multiple comparison test. Data are presented as mean ± s.e.m. ^*^ *p* < 0.05, ^**^ *p* < 0.01; ns: no significant difference.

We conducted imaging analysis on brain slices from Thy1‐YFP‐H transgenic mice following seven days of optogenetic activation of the SSp‐bfd. Note that these Thy1‐YFP‐H mice were previously validated through ofMRI experiments to be able to induce traveling waves with optogenetic activation of the SSp‐bfd. Our findings demonstrate that the same stimulation parameters enable reliably repeated induction of traveling waves (Figure S1, Supporting Information), ensuring that 7 days of optogenetic stimulation produced one wave per day in these mice. Interestingly, mice that underwent traveling waves exhibited a significant increase in dendritic spine density in the SSp‐bfd (*p* = 0.0492) and primary visual cortex (VISp; *p* = 0.0254) compared to those without traveling waves (**Figure** **5**B,C). These regions are located along the propagation pathway of the CSD‐like traveling waves (Figure 5A). In contrast, there was no significant difference in dendritic spine density between the two groups in the ventral tegmental area (VTA; *p* = 0.59) and dorsal raphe nucleus (DRN; *p* = 0.3251) (Figure 5D,E), which are not located within the propagation pathway of CSD‐like traveling waves (Figure 5A). These results showed that CSD‐like traveling waves promote synaptic plasticity changes in brain regions along their propagation pathway.

Changes in synaptic plasticity are commonly believed as a cellular substrate for learning and memory.^[^ ^66^ ^]^ To test this, we assessed the behavioral performance of another cohort of mice in the Y‐maze and novel object recognition tests following optogenetic activation of the SSp‐bfd. Similarly, these mice were screened using of MRI experiments to confirm the successful induction of traveling waves. No significant differences were observed in the Y‐maze alternation performance (main effect of group: *F_(2,36)_* = 0.1071, *p* = 0.8988; Figure S11A, Supporting Information) or the object recognition preference in the novel object recognition test (*p* = 0.2325, Kruskal‐Wallis test; Figure S11B, Supporting Information).

Previous studies have shown that an excessive increase in dendritic spine density is linked to anxiety‐like behaviors.^[^ ^67^, ^68^, ^69^ ^]^ Meanwhile, the high prevalence of anxiety in patients with CSD‐related disorders (e.g., migraine) suggests that CSD‐like traveling waves may contribute to anxiety‐like behaviors.^[^ ^70^ ^]^ To test this hypothesis, we performed a series of behavioral tests to assess anxiety levels in mice with traveling waves (Figure 5F). Compared to mCherry controls and mice without traveling waves, mice with traveling waves spent significantly less time in the center area during the open field test (main effect of group: *F_(2,36)_* = 5.287; *p* = 0.0097; Figure 5G,H, left). No significant difference was observed in general locomotor activity across groups (main effect of group: *F_(2,36)_* = 0.1077; *p* = 0.8982; Figure 5H, right). Moreover, CSD‐like traveling wave propagation significantly increased the latency to food in the novelty‐suppressed feeding test (main effect of group: *F_(2,18)_* = 7.09; *p* = 0.0054; Figure 5I, left), but did not affect the food consumption in mice (*F_(2,18)_* = 0.0661; *p* = 0.9363; Figure 5I, right). In the light‐dark box test, mice with traveling waves significantly increased entries into the dark box than those without traveling waves (*p* = 0.0084, Kruskal–Wallis test; with CSD vs without CSD: *p* = 0.011; Figure S11C). Collectively, these results show that CSD‐like traveling waves increased dendritic spine density along the propagation pathways, in parallel with the expression of anxiety‐like behaviors in mice.

## 3 Discussion

Traveling waves dynamically coordinate spatiotemporal patterns of neural activity across diverse brain regions to regulate behaviors. Despite global coordination, previous studies have largely focused on the propagation properties of traveling waves within localized brain regions.^[^ ^2^, ^3^, ^5^ ^]^ Here, we reveal brain‐wide spatiotemporal propagation dynamics of traveling waves using ofMRI, exemplified by mPFC‐ and SSp‐bfd‐induced CSDs. Our findings reveal that cortical traveling waves propagate to widespread brain regions, extending beyond the ipsilateral cortex to the contralateral cortex, hippocampus, amygdala, striatum, and brainstem. The spatiotemporal properties of the traveling waves, including propagation speed, brain region activation, and involvement, vary upon their origins and induction order. Furthermore, we identified a specific pathway through which cortical traveling waves convey information to the striatum, where traveling waves ultimately terminate. Importantly, we found that traveling wave propagation influences neuronal plasticity and induces anxiety‐like behaviors in mice. Collectively, our results for the first time provide a whole‐brain view of the complete propagation process of individual traveling waves, revealing their origin‐ and induction order‐dependent properties and their previously unrecognized role in resuting in anxiety‐like behaviors.

A key aspect of our findings is the identification of brain‐wide spatiotemporal coordination of traveling waves between cortical and subcortical structures, particularly the hippocampus, striatum, amygdala, and brain stem. Consistent with prior studies, we observed traveling waves propagating through both hemispheres of the cerebral cortex and into the hippocampus.^[^ ^28^, ^29^, ^30^ ^]^ Notably, our results showed that mPFC‐induced but not SSp‐bfd‐induced traveling waves, propagate to the hippocampus, especially the CA1, CA2, and CA3 regions (Figure 3A,F), despite the closer proximity of SSp‐bfd to these regions. These findings suggest that traveling wave propagation may serve as an additional neural communication form for the prefrontal cortex and hippocampus. Moreover, our findings that mPFC‐ and SSp‐bfd‐induced traveling waves share a common cortico‐amygdala‐striatal pathway and terminate their propagation in the striatum address critical questions about the termination of cortical traveling waves and their communication routes to deep subcortical nuclei.^[^ ^71^ ^]^ These findings also suggest that traveling waves play a significant role in transmitting cortical information to the striatum, potentially offering a mechanism relevant to brain functions and disorders involving cortico‐striatal connectivity.^[^ ^63^ ^]^ Given that most previous studies have focused on traveling waves within localized brain regions, particularly the cortex, our results underscore the importance of brain‐wide traveling wave propagation, especially within deep subcortical structures.

Previous studies of traveling waves, whether generated spontaneously or induced by sensory stimulus, have largely been observational without clear identification of their origins.^[^ ^12^, ^15^, ^72^, ^73^ ^]^ As a result, it is unclear where these traveling waves begin, nor whether the propagation properties differ for traveling waves originating from distinct brain regions. Our findings demonstrate that traveling waves can be induced by optogenetic activation, with the induction probability influenced by complex factors, such as brain region (e.g., mPFC, 23%; SSp‐bfd, 61%), light frequency, and inter‐individual differences in the animals.^[^ ^47^ ^]^ The higher probability of CSD induction following optogenetic activation of the SSp‐bfd compared to the mPFC supports previous studies suggesting a higher susceptibility of the sensory cortex to CSD‐like traveling waves.^[^ ^48^, ^49^ ^]^ Although the underlying mechanisms require further investigation, the relative impairment of potassium clearance in the sensory cortex may contribute to its increased sensitivity.^[^ ^48^ ^]^ Our results illustrate the power of ofMRI in interrogating the complete propagation dynamics from induction to termination of traveling waves on the whole brain scale. Using the well‐established ofMRI approach,^[^ ^74^, ^75^ ^]^ we found that mPFC‐ and SSp‐bfd‐induced traveling waves exhibit distinct propagation patterns across the cortex. In detail, mPFC‐induced traveling waves propagate along the anterior‐to‐posterior direction in the ipsilateral cortex, while SSp‐bfd‐induced traveling waves propagate diffusely from the stimulation site. In particular, in brain regions situated between the mPFC and SSp‐bfd, the traveling waves displayed opposite propagation directions. Different traveling wave directions modulate distinct human memory processing,^[^ ^3^ ^]^ and alterations in wave direction are associated with disorders such as memory deficits.^[^ ^20^, ^21^, ^22^ ^]^ Our findings suggest that differences in propagation direction may stem, at least partially, from the different origins where traveling waves begin to spread. Moreover, we observed that mPFC‐induced traveling waves propagate more slowly, activate more brain regions, and persist longer than SSp‐bfd‐induced traveling waves, further underscoring the role of origin in shaping propagation properties. Our findings here emphasize the roles of the origins in determining the spatiotemporal propagation properties of traveling waves.

Our results showed that the brain regions in mice experiencing traveling waves had significantly increased dendritic spine density compared to those without induced traveling waves (Figure 5B–E). This suggests that traveling waves can reshape neuronal connectivity, which may have long‐term effects on neuronal communication and behavioral outcomes. Our results of behavioral tests showed that the mice experiencing traveling wave propagation exhibited anxiety‐like behaviors (Figure 5I–K). These findings align with prior observations that traveling waves coordinate neural activity across brain regions to influence behavioral outcomes.^[^ ^3^, ^5^, ^14^, ^61^ ^]^ Importantly, our study identifies a previously unknown role of traveling waves in driving anxiety‐like behaviors, shedding light on a heretofore‐uncovered mechanism for anxiety disorders.

In this study, we used ofMRI with a 1‐s temporal resolution to explore the spatiotemporal propagation of traveling waves across the whole brain, using CSD as an example. While certain propagation characteristics of CSD observed in this study (e.g., speed and duration) may not be generalizable to all traveling waves, they provide valuable insights, such as the involvement of multiple cortical and subcortical structures and the dependence of propagation properties on wave origin. Our data showed that the CSD‐like traveling waves induced by optogenetic activation of SSp‐bfd propagated toward the prefrontal regions along the cerebral cortex (Figure 3F), following a trajectory that mirrors the sensorimotor‐to‐transmodal functional gradient reported in previous fMRI studies.^[^ ^76^, ^77^, ^78^ ^]^ This correspondence suggests that CSD‐like waves may follow the intrinsic hierarchical organization of large‐scale brain activity. Future study is needed to investigate the relationship between cortical hierarchical organization and the spatiotemporal dynamics of this slow‐propagating CSD‐like traveling waves.

Another important implication of our work here is to provide a framework for real‐time induction and whole‐brain detection of CSD. Despite the immense potential of CSD to be a therapeutic target for associated disorders, the mechanisms underlying CSD, particularly its brain‐wide propagation, remain poorly understood due to technical limitations in studying such spontaneously propagating waves. Compared to the traditional methods of inducing CSD, such as KCl injection or electrical stimulation, optogenetics allows for real‐time, targeted induction of single or recurrent CSD episodes, and longitudinal studies of CSD over several months.^[^ ^79^ ^]^ Meanwhile, fMRI can track the brain‐wide spatiotemporal propagation paths of CSD on a sub‐millimeter spatial resolution in rodents,^[^ ^14^, ^74^, ^80^, ^81^ ^]^ overcoming the limited coverage and depth constraints of traditional electrophysiological recording, optical imaging, and photoacoustic imaging techniques.^[^ ^71^, ^82^, ^83^, ^84^, ^85^ ^]^ Our findings enhance the understanding of CSD mechanisms and related disorders by demonstrating that both mPFC‐ and SSp‐bfd‐induced CSD propagate beyond the bilateral cortex, reaching the hippocampus, and through the amygdala to the striatum, which has been described separately in previous observations.^[^ ^86^, ^87^, ^88^ ^]^ A previous study has indicated that preventing CSD from propagating into the subcortical striatum and hippocampus is a potential mechanism of migraine pharmacological treatment.^[^ ^86^ ^]^ By examining the brain‐wide propagation of CSD, especially in subcortical structures, our study may provide new insights into the mechanisms underlying CSD itself and CSD‐related disorders, facilitating the development of novel therapeutic strategies.

In summary, our study provides a brain‐wide spatiotemporal view of the complete process of traveling wave propagation by integrating optogenetics with whole‐brain fMRI, and further reveals that such CSD‐like traveling wave propagation increases dendritic spine density and results in anxiety‐like behaviors in mice. This work underscores the necessity of studying the coordination of traveling waves across the whole brain to understand their role in regulating behavior.

## 4 Experimental Section

### Animals

All animal procedures adhered to the regulations of the Administration of Affairs Concerning Experimental Animals (China) and were approved by the Southern Medical University Animal Ethics Committee (No. L2020025). Male C57BL/6J mice (≈7‐weeks‐old) were purchased from the Southern Medical University Animal Center (Guangzhou, China). Thy1‐YFP‐H transgenic mice (Jackson stock: #003782) were initially sourced from the Jackson Laboratory and bred in‐house. Mice were housed in temperature‐ and humidity‐controlled rooms with a 12‐h light‐dark cycle (lights on from 7:00 to 19:00) and were provided ad libitum access to food and water.

### Viruses

All viruses were obtained from Taitool Bioscience (China), including AAV2/9‐mCaMKIIα‐hChR2(H134R)‐mCherry‐WPRE‐pA (4.06 × 10^12^ particles/ml) and AAV2/9‐mCaMKIIα‐mCherry‐WPRE‐pA (1.60 × 10^13^ particles per mL).

### Stereotactic Injection and Optic Fiber Implantation

Stereotactic surgery and fMRI data acquisition followed a previously established protocol.^[^ ^74^ ^]^ Eight‐week‐old mice were anesthetized with 3.5% isoflurane and positioned in a stereotactic frame with their teeth secured with a bite bar and their head fixed by ear bars (RWD Life Science, China). Isoflurane was reduced and maintained at 1.5–2% throughout subsequent surgery, and the mice were placed on a heating pad to maintain physiological body temperature. Ophthalmic ointment was applied to prevent eye dryness. The hair on the top of the mice's head was shaved, the scalp was disinfected with 75% alcohol, and an incision of about 1cm was made along the middle. A small craniotomy was performed using a stereotactic drill. Virus injections were administered at coordinates based on the Paxinos and Franklin Mouse Brain Atlas: SSp‐bfd (AP: −1.00 mm, ML: +3.00 mm, DV: −1.75 mm) and mPFC (AP: +1.70 mm, ML: +0.35 mm, DV: −2.70 mm). A volume of 300‐nL of virus was injected using a 33‐gauge needle attached to a 5‐µL microsyringe (Hamilton, USA) and a microsyringe pump (Stoelting, USA). After injection, the needle was left in place for 10 min before being slowly withdrawn. Tissue glue (No.03‐396, DentKist, Korea) was applied to concrete the scalp. Mice were allowed to recover for at least three weeks post‐injection to ensure optimal ChR2 expression before optic fiber implantation.

To improve the signal‐to‐noise ratio and reduce imaging artifacts, custom‐made light‐angle plastic optical fibers (Numerical Aperture = 0.5, diameter = 220 µm) were implanted. These fibers allowed the head cryogenic coil to be positioned closer to the mouse head, minimizing susceptibility differences compared to commonly used quartz fibers.^[^ ^74^ ^]^ The custom‐made fibers were wrapped in black opaque heat‐shrinkable sleeves to prevent unwanted visual stimulation during optogenetic experiments. Before implantation, the fiber tip was clipped to create an angled surface to facilitate insertion and thus reduce brain tissue damage. The fibers were implanted above the cells infected with ChR2. Coordinates for fiber implantation in the optogenetic activation of SSp‐bfd were AP: −1.00 mm, ML: +3.00 mm, DV: −1.65 mm, and for the mPFC: AP: +1.70 mm, ML: +0.35 mm, DV: −2.60 mm. Dental cement was used to secure the right‐angle fibers to the mice's skull. After implantation, mice were monitored for recovery from anesthesia and housed individually to prevent fiber damage due to mutual grooming or chewing. Mice were allowed to recover for a week in their home cages before undergoing optogenetic fMRI experiments.

### Animal Preparation and Data Acquisition for Optogenetic fMRI Experiments

Mice were initially anesthetized with 3.5% isoflurane, followed by an intraperitoneal injection (i.p.) of 0.02 mg kg^−1^ dexmedetomidine after 5 min. To minimize discomfort, 2.5% lidocaine drops were applied to the cords. Mice were endotracheally intubated and tightly fixed on the animal bed with a bite bar, ear bars, and a home‐made 3D‐printed mouse breathing mask compatible with endotracheal intubation under the Bruker cryogenic coil (https://www.bio‐protocol.org/). Mice were connected to a small animal ventilator (TOPO, Kent Scientific) and mechanically ventilated at a rate of 80 breaths per minute, with a respiration cycle of 25% inhalation and 75% exhalation. Muscle relaxant, pancuronium bromide (TargetMol), was injected i.p. at a dose of 0.2 mg kg^−1^ to reduce the motion of mice. Ophthalmic ointment was applied to protect the eyes. Isoflurane was reduced and kept at 0.4%, with a continuous subcutaneous dexmedetomidine infusion (0.04 mg kg^−1^ h^−1^) to maintain sedation. Throughout the experiment, rectal temperature was maintained at 37 ± 0.1 °C using an MR‐compatible heater system (Heater system, Small Animal Instruments). Animal preparation took ≈10–15 min.

All MRI experiments, except for ofMRI experiments in female mice (9.4 T MRI, cryogenic coil, and the same scan parameters), were conducted in a Bruker 7T MRI scanner equipped with a mouse head cryogenic coil (Bruker, Germany). High‐resolution T2‐weighted anatomical images were acquired using a Turbo‐RARE sequence to validate the optical fiber placement and register the fMRI images with a field of view (FOV) = 16 × 16 mm^2^, matrix = 256 × 256, RARE factor = 8, repetition time (TR)/ echo time (TE) = 2500 ms/35 ms, slice thickness = 0.4 mm. The magnetic field homogeneity was optimized using the MAPSHIM protocol with previously acquired B0 field maps. Functional MRI images were then acquired using a single‐shot Gradient‐Echo‐Planar‐Imaging (GE‐EPI) sequence with FOV = 16 × 16 mm^2^, matrix = 64 × 64, flip angle = 54.7°, slice thickness = 0.4 mm, TR/TE = 1000 ms/15 ms, dummy scans = 10, 420‐900 time points.

An Arduino programming board synchronized the MRI scanner with laser transmitters (SDL‐473‐100MFL, Shanghai Dream Lasers Technology Co., Ltd, China). For optogenetic stimulation, the laser transmitters were placed outside the magnet with a 5 to 10‐m optical patch cable used to deliver blue light (473 nm) into the injected site. Laser power was measured by a power meter (PM100D and S142C, Thorlabs, USA).

### Traveling Wave Induction Experiments

To investigate whether 40 Hz optogenetic activation of the mPFC could induce CSD‐like traveling waves, a total of 69 ChR2‐expressing mice received a single trial of 10‐s pulse trains of light stimulation at 40 Hz (3.5 mW, 30% duty cycle). We used the Paravision 360 (Bruker, Germany) or ImageJ software to visually inspect whether optogenetic stimulation induced CSD‐like traveling waves, which are characterized by sequential activation of brain regions and appear as a cortical wave of BOLD signal changes propagating across different regions over time (Video S1, Supporting Information). Moreover, we also checked the presence of CSD‐like traveling waves by calculating brain activation maps. In mice with induced CSD‐like traveling wave, distinct brain regions showed sequential activation at different time points after the termination of optogenetic stimulation. Among the 69 mice, optogenetic activation of the mPFC induced traveling waves in 16 mice.

After 1–3 days of recovery, these 16 mice underwent a randomized multi‐frequency stimulation of mPFC. Each mouse received 9 to 10 trials of optogenetic stimulation, for a total of 156 trials, in which mice received a single stimulation at one of six randomly selected frequencies (2, 10, 25, 40, 80, or 125 Hz) on each trial. Each trial was visually inspected to determine whether the CSD‐like traveling wave was induced. Subsequently, the activation map of each trial was calculated to verify whether optogenetic activation of mPFC induced traveling waves in that trial. After a week of recovery, seven of these mice were used to repeat the above experiment to verify the reproducibility of optogenetically induced traveling waves.

In the multi‐frequency stimulation experiment, if the 40 Hz optogenetic stimulation is the first trial, the traveling wave induced in this trial is defined as the “First‐induced wave.” Otherwise, if the mouse has already experienced one or more traveling wave propagations before the 40 Hz stimulation within a single experiment, the wave induced by the 40 Hz stimulation is considered a recurrent traveling wave (referred to as the “Re‐induced wave”).

In the experiment involving optogenetic activation of SSp‐bfd, each mouse received 1‐2 trials of 40 Hz optogenetic stimulation. If any trial induced a traveling wave, the mouse was considered capable of generating a traveling wave in response to 40 Hz optogenetic activation of SSp‐bfd. The traveling wave induced by the first 40 Hz stimulation was defined as the “First‐induced wave,” while a wave that could still be induced after the first induction was defined as a recurrent wave (referred to as the “Re‐induced wave”).

### Optogenetic fMRI Data Preprocessing

After converting the data to NIfTI format, the mouse brain was extracted using an in‐house automatic brain extraction algorithm implemented in Python.^[^ ^89^ ^]^ The effect of skull stripping was validated using ITK‐SNAP software (http://www.itksnap.org/). Subsequently, all preprocessing procedures were conducted following a standardized protocol in SPM12 (Wellcome Department of Imaging Neuroscience, University College, London, UK) within MATLAB 2017b (MathWorks, USA). Specifically, functional images were slice‐timing corrected, realigned to the mean of the first volume, and registered to the corresponding anatomical images. The 3D Allen Mouse Brain Common Coordinate Framework, V3 (CCF v3) (http://help.brain‐map.org/) was downsampled into both a high‐resolution space (114 × 80 × 132 voxels at 0.14 × 0.13 × 0.15 mm^3^) and a low‐resolution space (114 × 80 × 36 voxels at 0.14 × 0.13 × 0.4 mm^3^). Anatomical images were then registered to the CCF v3 template via affine registration, with the transformation matrix subsequently applied to the functional images. Functional images were resliced into a common group space similar to the down‐sampled CCF v3 templates. High‐resolution functional images were used for displaying 3D activation maps, while low‐resolution functional images were employed for calculating 2D activation maps. The twelve head motion parameters—including the roll, pitch, yaw, and translation in 3D, along with their first derivative—were regressed out from the BOLD signal of each voxel. The BOLD signal was then band‐pass filtered to 0.001–0.1 Hz, followed by spatial smoothing with a 0.2 mm isotropic Gaussian kernel.

### Definition of Regions of Interest (ROIs)

A total of 68 ROIs were defined based on the Allen Mouse Brain Atlas (http://connectivity.brain‐map.org/), encompassing the cerebral cortex (53 ROIs), cerebral nuclei (5 ROIs), brain stem (8 ROIs), and cerebellum (2 ROIs). See Table S1 (Supporting Information) for the details.

### Hemodynamic Response Function (HRF)

In trials with optogenetically‐induced traveling waves, the HRF of the BOLD signal exhibited distinct characteristics compared to the canonical human HRF in SPM12. Consequently, a data‐derived HRF was established to generate activation maps in these trials. A total of 1242 BOLD signals (10‐s optogenetic stimulation) were averaged to fit the HRF using a double gamma variate function. The parameters of the HRF function (the spm‐hrf function in the SPM12 toolbox in MATLAB) were 29.5, 25.5, 10, 1.75, 0.63, 0, and 100. This data‐derived HRF was convolved with a boxcar function and applied in a general linear model (GLM) analysis to generate first‐level activation contrasts.

### Calculation of fMRI Activation Maps

To calculate activation maps during optogenetic stimulation for control and the group without traveling waves, GLM analysis with default HRF was used to generate response maps for individual EPI scans in SPM12. The default HRF was convolved with a slide‐window design matrix (10‐s window width, sliding in steps of 5 TRs) as the explanatory model to obtain the dynamic activation maps. A voxel‐wise one‐sample *t*‐test (against zero) was performed to identify activated voxels with a statistical threshold of false discovery rate (FDR)‐corrected *p* < 0.01.

For assessing activation patterns during traveling wave propagation, the data‐derived HRF was convolved with a slide‐window design matrix (10‐s window width, sliding in steps of 5 TRs) as the explanatory model to obtain the dynamic activation maps. Other default options in SPM12, such as auto‐correlation modeling and a high‐pass filter (128 s), were used. A one‐sample *t*‐test (against zero) was performed to identify activated voxels, with voxels passing the thresholds of FDR‐corrected *p *< 0.01 deemed statistically significant. To enhance the visualization of traveling wave propagation, *t*‐maps were gamma‐transformed (γ = 3) and visualized in 3D using ImageJ (http://rsb.info.nih.gov/ij/). We also extracted voxel‐wise *t*‐values of the dynamic activation maps within 68 ROIs of the bilateral brain to characterize the spatiotemporal activation of traveling waves. Only |*t*‐value| > 5 is shown, as it corresponds to a Family‐Wise Error (FWE)‐corrected *p* < 0.05. The mean beta value of each ROI was absolutized, normalized (divided by the average beta value of the stimulation ROI), and displayed on a logarithmic scale to characterize the brain responses (Figure 3C,H).

Time series of two ROIs on the cortex at a distance of 2.2923 mm were extracted. The time interval between the negative peaks of the BOLD signals of the ROIs was considered to be the propagation time from one ROI to the other. The propagation speed of the traveling wave was calculated as the distance of 2.2923 mm divided by the time interval between the two ROIs.

### Logistic (Sigmoid) Function Fitting

The relationship between optogenetic stimulation frequency (*f*) and the proportion of traveling wave induction (*P*) was modeled using a Logistic function:

(1) $$P\left(f\right)=\frac{1}{1+e^{-k\left(f-f_{50}\right)}}$$

where:*k* detemines the slope of the transition. *f*_50_ (threshold frequency) indicates the point where the traveling wave induction probability significantly increases.

Parameters (*k*,  *f*_50_) were estimated via nonlinear least‐squares fitting (initial guesses: *k* = 0.1,  *f*_50_ = 50). The *R* ^2^ was calculated to quantify the variance explained by the model. In this study, the observed data are: *f* = [2,  10,  25,  40,  80,  125] (Hz), $P\left(f\right)=\left[0,\frac{2}{25},\frac{6}{24},\frac{18}{31},\frac{29}{32},\frac{22}{24}\right]$, and the parameters were *k* = 0.091,  *f*_50_ = 37 *Hz*,  *R* ^2^ = 0.9828.

### Cross‐Correlation Analysis

To trace the propagation sequence of CSD‐like waves, we performed a voxel‐wise cross‐correlation analysis. First, we identified all voxels activated by the CSD‐like wave based on the activation map (|*t*‐value| > 5, corresponding to FWE‐corrected *p* < 0.05), and then extracted the BOLD signals of all voxels. Then, the cross‐correlation analysis between the data‐derived HRF and the BOLD signal in individual voxels was calculated. The time lag corresponding to the maximum correlation coefficient is determined to be the moment that the CSD‐like traveling wave reached this voxel. The voxels are then sequentially aligned in order of time delay to show the propagation of the CSD‐like wave.

### Calculation of Structural Connectivity

Structural connectivity data were sourced from the axon projection tracer experiment in the Allen Mouse Connectivity Atlas (http://connectivity.brain‐map.org/; Experiment # 157556400). The virus was injected in the right infralimbic area (a mPFC subregion). To quantify structural connectivity, the projection volume in each of the 68 ROIs was normalized by dividing by the right infralimbic area projection volume, and the results were presented on a logarithmic scale.

### Behavioral Assays

Mice were implanted with optical fibers over infected cells in the ipsilateral SSp‐bfd region. One week after implantation, an ofMRI experiment was conducted to assess whether optogenetic activation induces CSD‐like traveling waves in mice, with 10‐s, 40 Hz, 30% duty cycle, and 3.5 mW blue light. The positions of the fibers were verified using T2‐weighted high‐resolution anatomical images, and viral expression was confirmed through confocal imaging. Mice were handled for 5 min daily over three days and transported to the testing room 1 day before behavioral tests to habituate the experimental environment. Behavioral tests were performed between 1:00 p.m. and 6:00 p.m. by experimenters who were blinded to group allocations. To diminish odor cues, 70% ethanol solution was used to clean the experimental apparatus between trials. Mice received 10‐s blue light (473 nm, 40 Hz, 30% duty cycle, 3.5 mW) stimulation, and were then returned to their home cages for a 30‐min rest period before undergoing behavioral tests.

### Open Field Test

Mice were placed in the center of an open field apparatus (length × width × height = 40 × 40 × 30 cm^3^) consisting of a 16 × 16 infrared grid. They were allowed to explore freely for 5 min. The cumulative time spent in the central 8 × 8 grid region (20 × 20 cm^2^) and the total distance traveled were recorded and analyzed using Fusion 6.47 software in SuperFlex Open Field System (USA).

### Light‐Dark Box Test

On one side of the open field apparatus (40 × 40 × 30 cm^3^), a black Plexiglas box (40 × 20 × 12 cm^3^) was placed to divide the chamber into two equal compartments. Mice were placed in the center of the light compartment and allowed to explore freely for 5 min. The latency to first enter the dark compartment, total entries to the dark compartment, and cumulative time spent there were recorded and analyzed using Fusion 6.47 software in SuperFlex Open Field System (USA).

### Novelty‐Suppressed Feeding Test

After food deprivation for 24 h, mice were placed into one of the corners of a brightly lit open arena (50 × 50 × 50 cm^3^) containing a single food pellet placed in the centre. The latency to begin feeding within the 5‐min period was recorded and analyzed using EthoVision 11.0 software (Noldus, Netherlands). After testing, mice were returned to their home cages, and food consumption was measured within the following 5 min.

### Y‐Maze Spontaneous Alternation Test

The Y‐maze apparatus consisted of three identical black arms (30 × 10 × 20 cm^3^ per arm), arranged at a 120° angle. Mice were gently placed in the center of the apparatus and allowed free access to all three arms for 8 min. The total number of arm entries and the sequence of entries were recorded to calculate the percentage of spontaneous alternations. Alternation was defined as consecutive entry into each arm, and alternation percentage was calculated as: (number of actual alternations/ maximum possible alternations) × 100.

### Novel Object Recognition Test

The novel object recognition test involved three phases: habituation, training, and testing. During habituation, mice were placed in an empty Plexiglas rectangular cage (25 × 25 × 25 cm^3^) and allowed to explore for 10 min. In the training phase, 24 h later, two identical objects were placed at an equal distance in the cage, and the mice were allowed to explore for 10 min. The objects used were either Falcon tissue culture flasks or towers of Lego bricks, chosen randomly to avoid bias. In the testing phase, 24 h after training, one of the familiar objects was replaced by a novel object. Mice received 10‐s optogenetic stimulation followed by 30 min of rest in their home cage before being placed back in the open field arena o to explore the familiar and novel objects for 5 min. Time spent exploring each object was recorded, with investigation behaviors within 2.5 cm around the object considered exploration behaviors, such as sniffing or touching the object. The preference for the novel object was assessed using the preference index, calculated as: Preference index = (time spent exploring the novel object)/(time spent exploring the novel object + time spent exploring the familiar object). Data were analyzed using EthoVision software (Noldus, Netherlands).

### Elevated Plus Maze

The apparatus for the elevated plus maze test consisted of two open arms (30 × 5 × 0.5 cm^3^) and two vertical closed arms (30 × 5 × 15 cm^3^) crossing each other, with the connecting part forming a central platform (5 × 5 cm^2^). The apparatus was elevated 50 cm above the ground. Mice were gently placed on the central platform and allowed to explore freely for 5 min. Behavioral performances were tracked, and the time spent in the open and closed arms was recorded using EthoVision 11.0 software (Noldus, Netherlands).

### Immunohistochemistry and Histology

Mice were deeply anesthetized with isoflurane and transcardially perfused with saline, followed by 4% paraformaldehyde (PFA). Brains were extracted, postfixed in 4% PFA overnight at 4 °C, and subsequently dehydrated in a 30% sucrose solution until isotonic. After three days, 40‐µm coronal brain slices were obtained using a freezing microtome (Leica, CM1950, Germany) at −20 °C. The brain slices were washed three times with phosphate‐buffered saline (PBS, 0.1 M, PH 7.4). The 4′,6‐Diamidino‐2‐phenylindole (DAPI) was used for fluorescent nuclear counterstaining. The brain slices were mounted on glass slides and visualized using a Nikon C2 confocal microscope (Japan). To verify the expression of viruses, the brain slices of SSp‐bfd and mPFC were imaged by a Nikon C2 confocal microscope (Japan) with a 20× objective.

For quantitative analysis of dendritic spine density, secondary and tertiary apical dendritic segments parallel to the section surface were imaged using a Nikon C2 confocal microscope (Japan) with a 60× oil objective. Z‐stack imaging was performed with 0.1‐µm step intervals along the *z*‐axis. The images were imported, calibrated, and manually traced using Imaris 8.1 software.

### Statistical Analysis

Statistical analyses were conducted using SPSS 22.0. Two‐tailed two‐sample *t*‐tests were used to evaluate differences between two groups with normally distributed data, while the Mann–Whitney U test was applied to compare the differences in two groups that are heterogeneous in terms of variance. For comparisons among multiple groups, one‐way ANOVA followed by Tukey's multiple comparison test was used. Kruskal‐Wallis test followed by Dun's multiple comparisons test was applied to compare the differences in three groups that are heterogeneous in terms of variance. Statistical significance was set at ^*^ *p* < 0.05, ^**^ *p* < 0.01, ^***^ *p* < 0.001, and ^****^ *p* < 0.0001, and ns represents no significant difference.

## Conflict of Interest

The authors declare no conflict of interest.

## Author Contributions

J.L., J.‐W.M., and X.W. contributed equally to this work. Y.F., X.C., Q.H., J.L., J.‐W.M., and X.W. performed Conceptualization. J.L., Z.A., Y.M., and S.T. performed fMRI experiments. J.L. and C.T. performed fMRI data analysis. J.‐W.M. and Q.W. performed surgery. J.‐W.M., P.‐L.K., J.R., C.‐L.L, Y.M., and S.T. performed behavioral experiments and analysis. J.‐W.M., L.D., and Y.M. performed immunofluorescence experiments. J.L. and X.W. performed the results interpretation. J.L. and J.‐W.M. performed visualization. J.L., J.‐W.M., and X.W. wrote the original draft. J.L., J.‐W.M., X.W., Q.H., E.X.W., X.C., and Y.F. wrote, reviewed, and edited.

## Supporting information



Supporting Information

Supplemental Video 1

Supplemental Video 2

Supplemental Video 3

## Data Availability

The data that support the findings of this study are available from the corresponding author upon reasonable request.
