# Supplementary material for: Brain‐Wide Spatiotemporally Distinct Traveling Waves Drive Anxiety‐Like Behaviors in Mice
Source: Adv Sci (Weinh). 2025 Jul 29;12(40):e11867. doi: 10.1002/advs.202411867 (PMC12561208; doi:10.1002/advs.202411867)
Supplement: Supplementary file 1 — Supporting Information [file ADVS-12-e11867-s001.docx]

Supporting Information

Brain-wide spatiotemporally distinct traveling waves drive anxiety-like behaviors in mice

*Jiaming Liu, Jia-Wen Mo, Xunda Wang, Yinuo Ma, Shile Tian, Qi Wang, Peng-Li Kong, Ziqi An, Li Ding, Jing Ren, Cheng-Lin Lu, Chuanjun Tong, Ed X. Wu, Qiu-gen Hu*, Xiong Cao*, Yanqiu Feng**


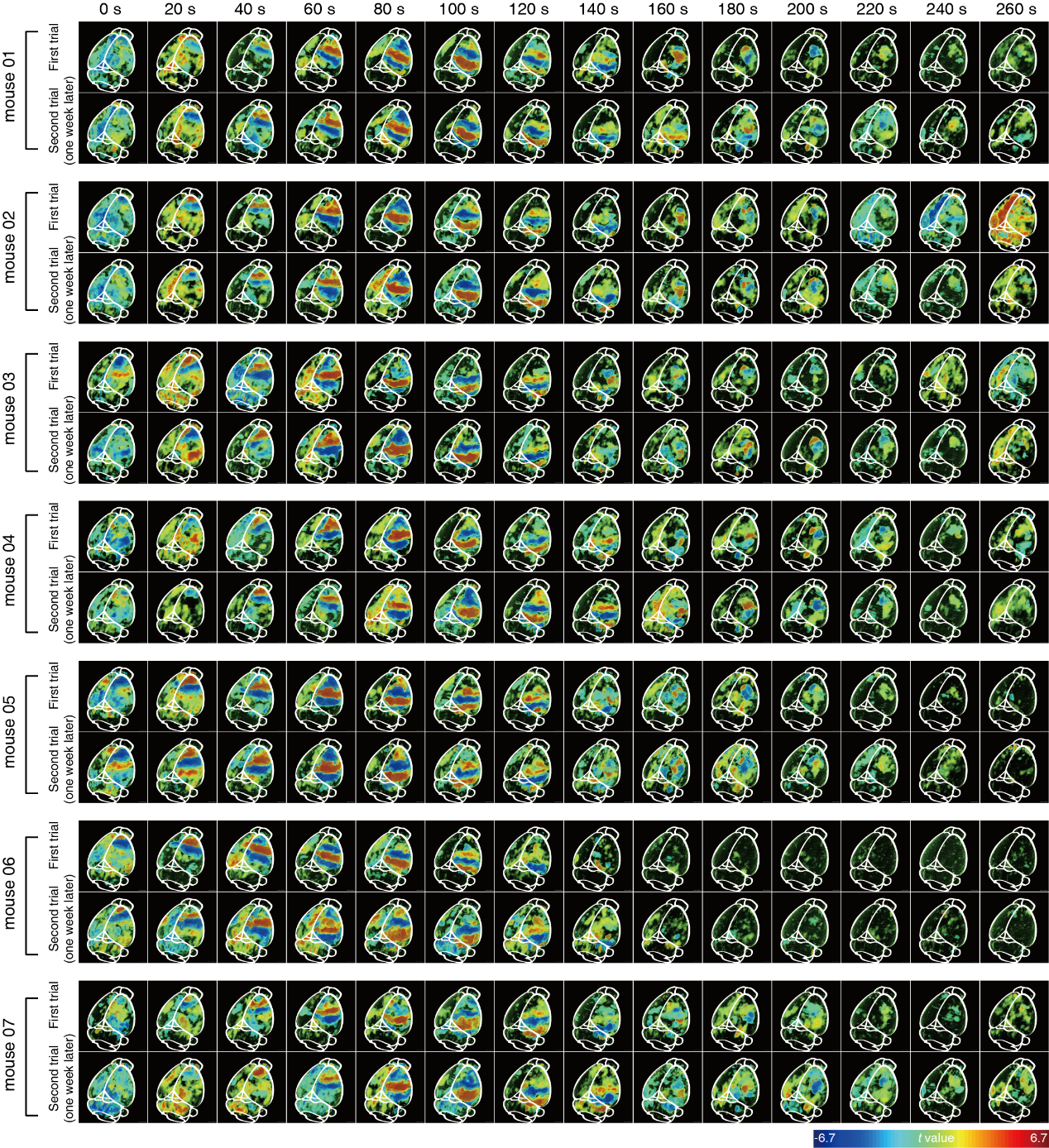


**Figure S1.** Reproducibility of optogenetic-induced cortical traveling waves. Seven mice that induced traveling waves via 10-second of 40 Hz, 3.5 mW optogenetic activation of the mPFC successfully induced traveling waves again after one week using identical stimulation parameters.


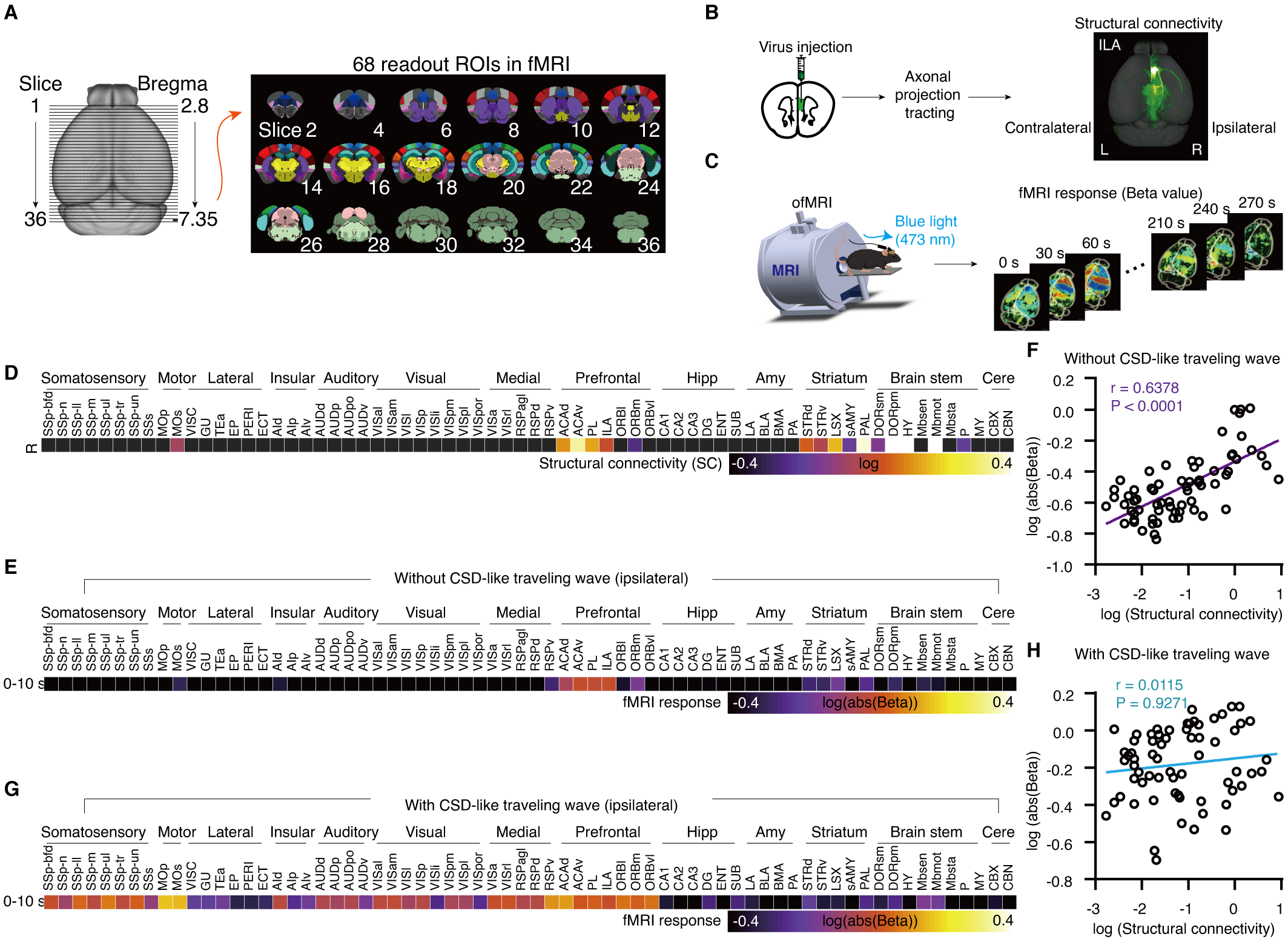


**Figure S2.** Traveling wave propagation is largely unconstrained by axonal projections. A) Sixty-eight ROIs were defined based on the Allen Mouse Brain Atlas. B) Axonal projection volumes imported from the Allen Mouse Brain Connectivity Atlas were used to quantify the structural connectivity of the mPFC (<http://connectivity.brain-map.org/>; Experiment # 157556400). C) Beta values from the general linear model analysis were applied to quantitative fMRI responses. D) Structural connectivity in ipsilateral 68 ROIs (log scale) was represented using color coding. Structural connectivity strength was calculated as the summed axonal projection volumes normalized by the virus injectional volume in the mPFC. E) fMRI responses in 68 ipsilateral ROIs (log scale, normalized by the beta value in ILA) of mice without mPFC-induced CSD-like traveling waves (22 mice, 29 trials). F) Correlation analysis between structural connectivity and fMRI responses during optogenetic stimulation (0-10 seconds) in the mice without traveling waves. G) fMRI responses in mice with mPFC-induced traveling waves (23 mice, 31 trials). H) Correlation analysis between structural connectivity and fMRI responses during optogenetic stimulation (0-10 seconds) in mice with traveling waves. See Table S1 for details on ROI definitions and abbreviations.


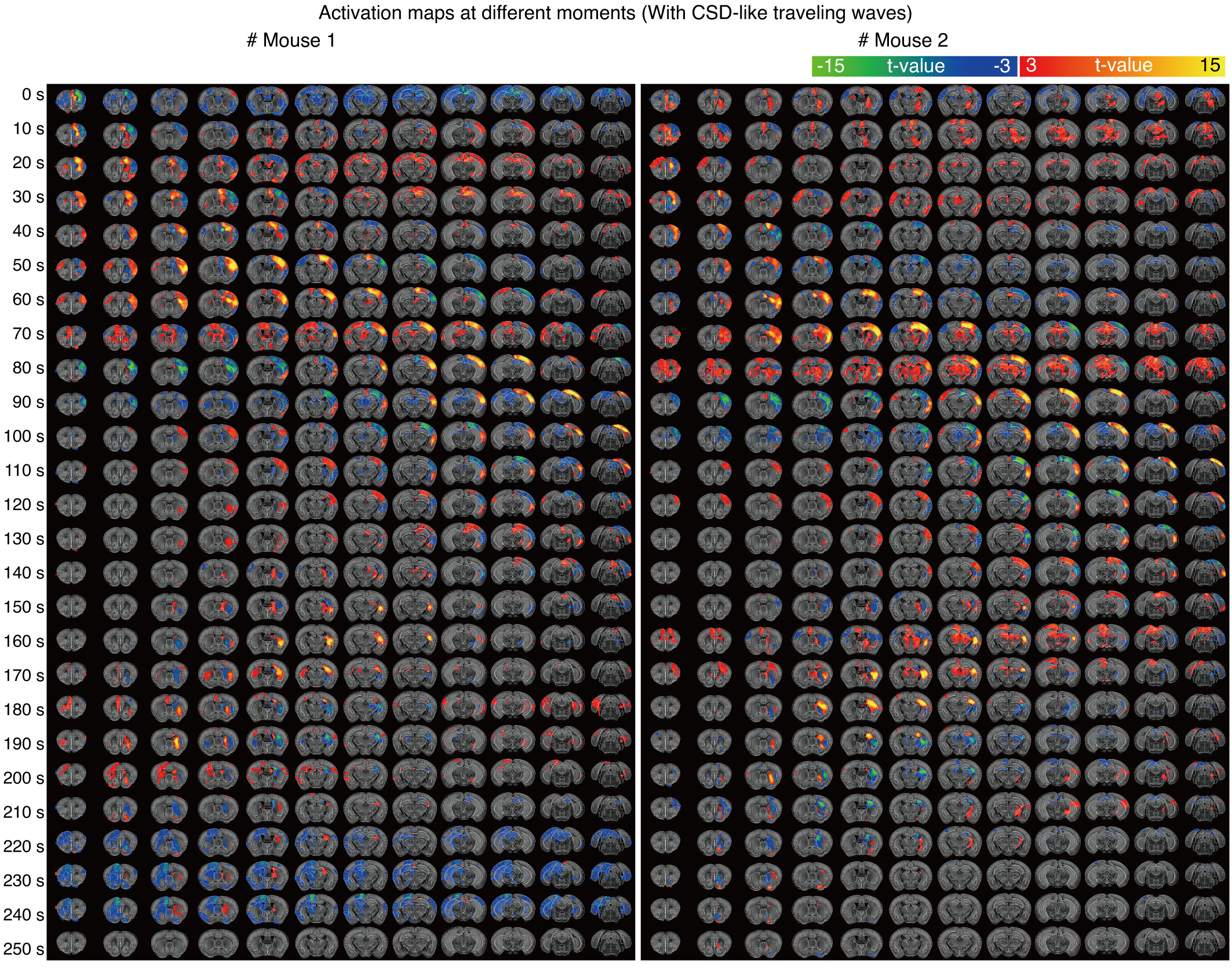


**Figure S3.** Activation maps at different time points for two representative mice with traveling waves, following 10-second optogenetic activation of the mPFC (40 Hz, 3.5 mW, 30% duty cycle, and 473 nm blue light).


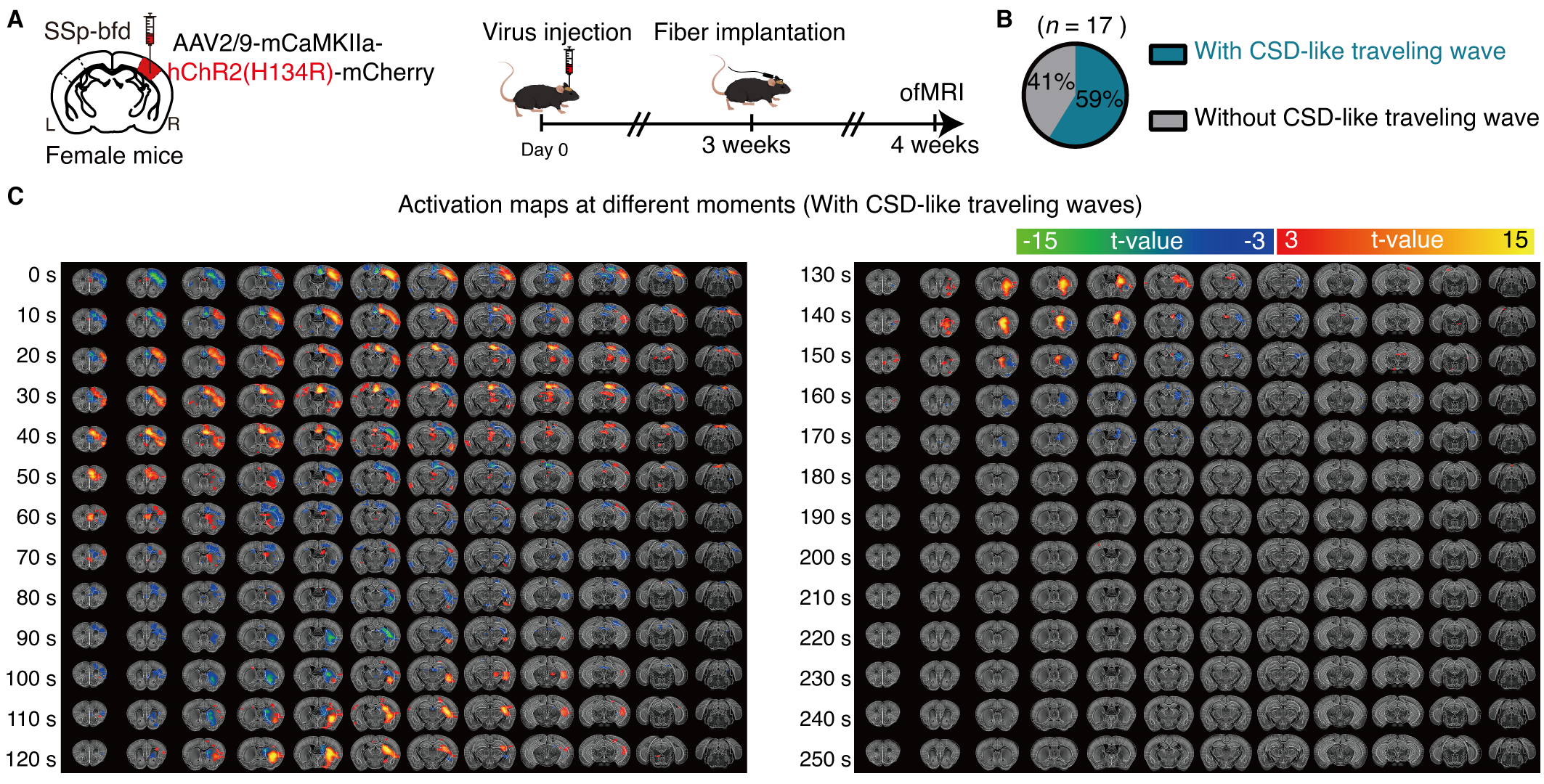


**Figure S4.** Optogenetic activation of SSp-bfd in female mice induces CSD-like traveling waves. A) Schematic illustration depicting the timeline of unilateral viral injection, optical fiber implantation, and ofMRI experiments. B) Percentage of CSD-like traveling waves induced by 40 Hz optogenetic activation of the SSp-bfd in female mice. C) Activation maps at different time points for a representative female mouse exhibiting SSp-bfd-induced traveling waves, following 10-second optogenetic stimulation (40 Hz, 3.5 mW, 30% duty cycle, and 473 nm blue light).


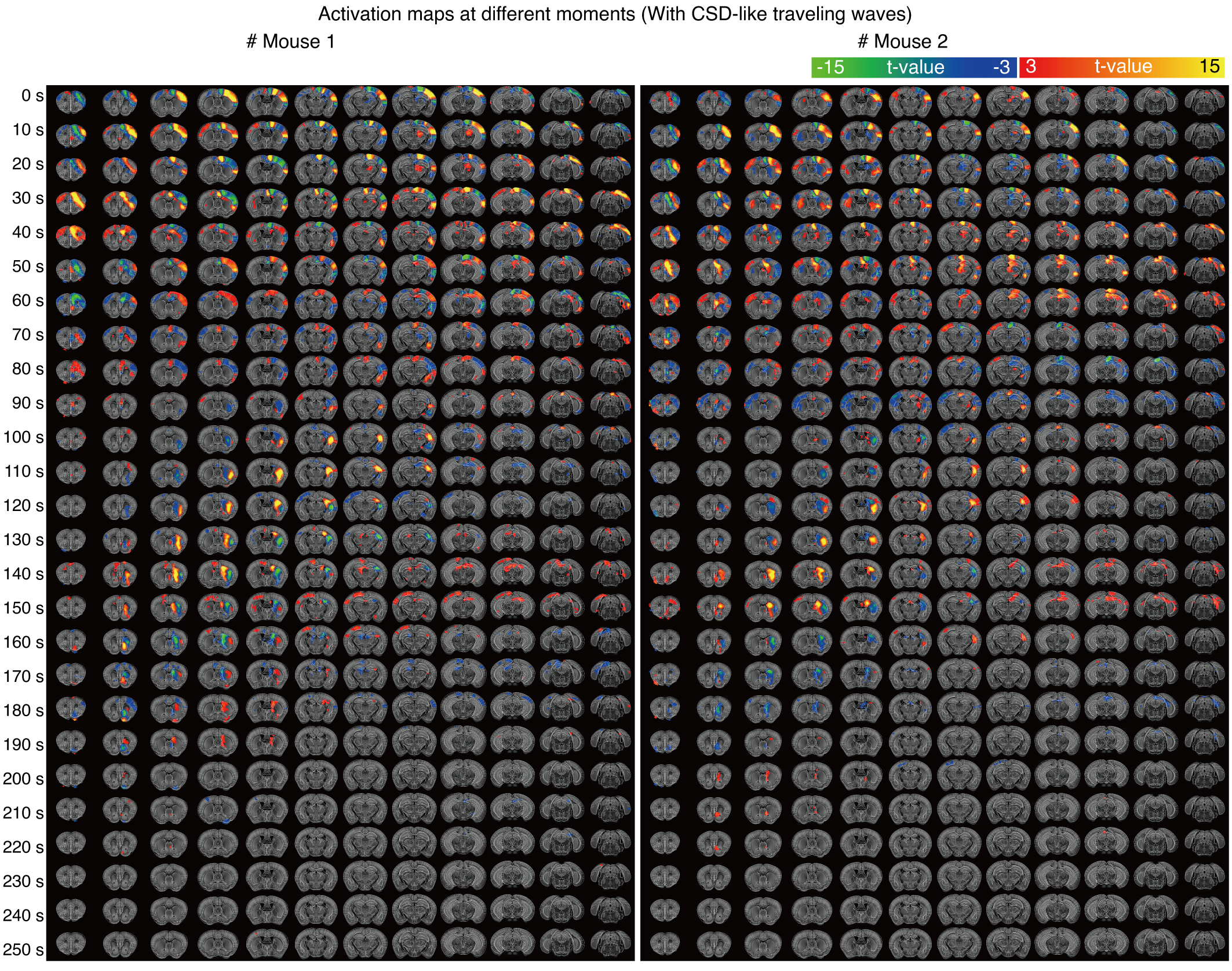


**Figure S5.** Activation maps at different time points for two representative male mice exhibiting traveling waves, following 10-second optogenetic stimulation of the SSp-bfd (40 Hz, 3.5 mW, 30% duty cycle, and 473 nm blue light).

**
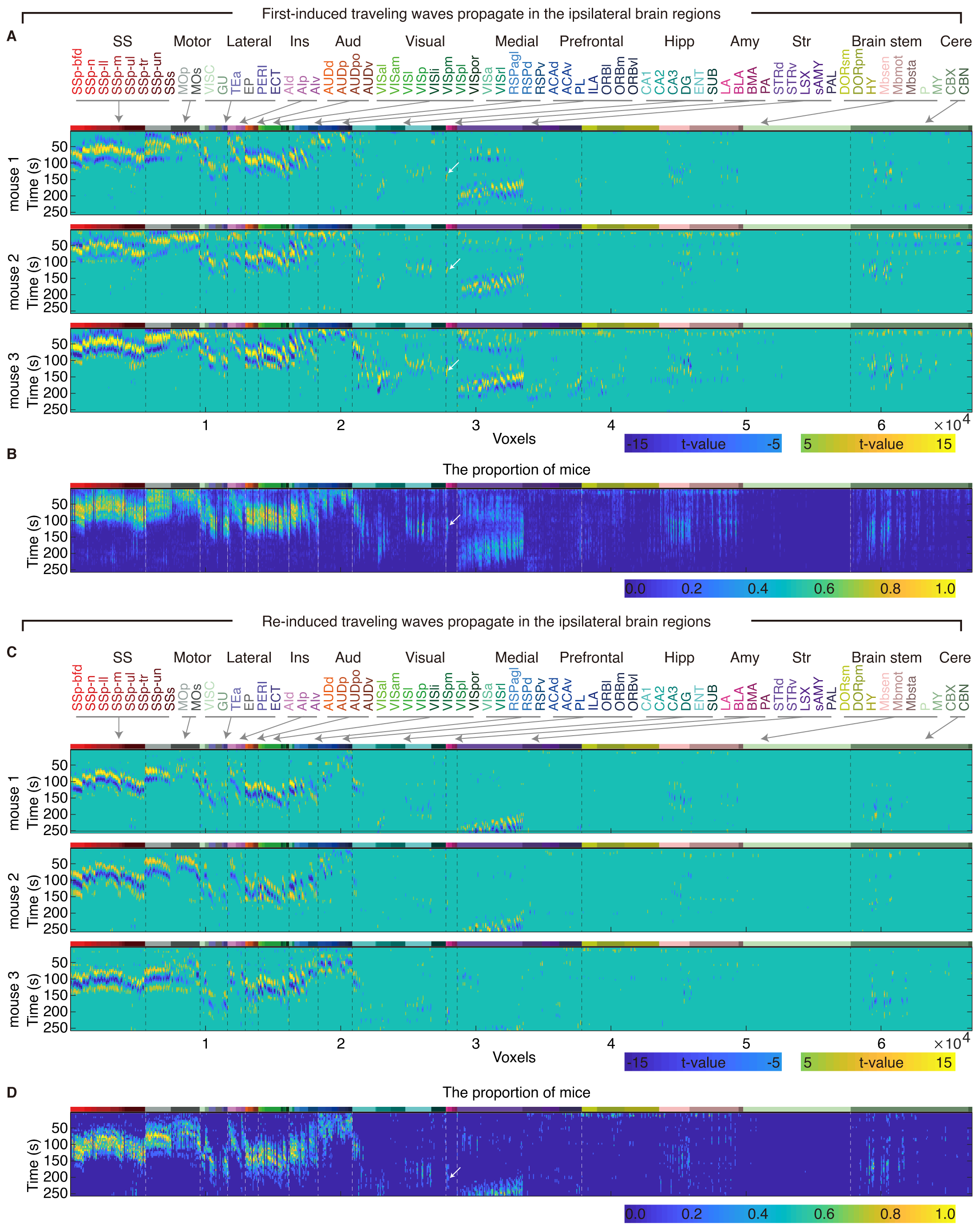
**

**Figure S6.** Voxel-wise spatiotemporal activation of first- and re-induced traveling waves induced by optogenetic activation of the mPFC. A) Spatiotemporal activation patterns in the ipsilateral brain regions in three representative mice undergoing the first-induced traveling wave. B) The proportion of mice activated by the first-induced traveling waves at different times in the ipsilateral brain regions (*n* = 26 mice). C) Spatiotemporal activation patterns in the ipsilateral brain regions in three representative mice undergoing the re-induced traveling wave. D) The proportion of mice activated by the re-induced traveling waves at different times in the ipsilateral brain regions (*n* = 5 mice). White arrows emphasize that the lateral amygdala (LA) is activated first before the dorsal striatum (STRd) is activated.

**
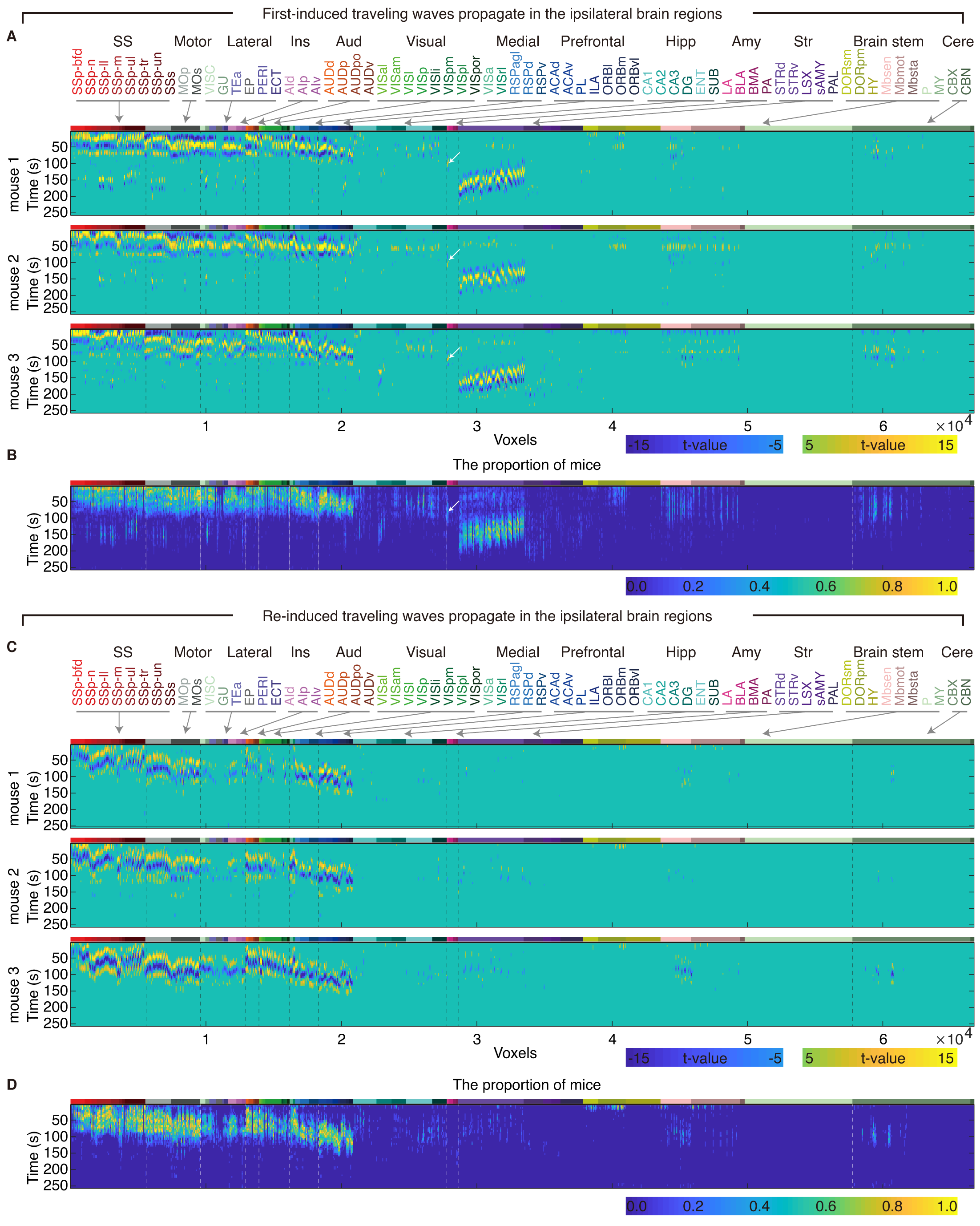
**

**Figure S7.** Voxel-wise spatiotemporal activation of first- and re-induced traveling waves induced by optogenetic activation of the SSp-bfd. A) Spatiotemporal activation patterns in the ipsilateral brain regions in three representative mice undergoing the first-induced traveling wave. B) The proportion of mice activated by the first-induced traveling waves at different times in the ipsilateral brain regions (*n* = 19 mice). C) Spatiotemporal activation patterns in the ipsilateral brain regions in three representative mice undergoing the re-induced traveling wave. D) The proportion of mice activated by the re-induced traveling waves at different times in the ipsilateral brain regions (*n* = 10 mice). White arrows emphasize that the LA is activated first before the STRd is activated.


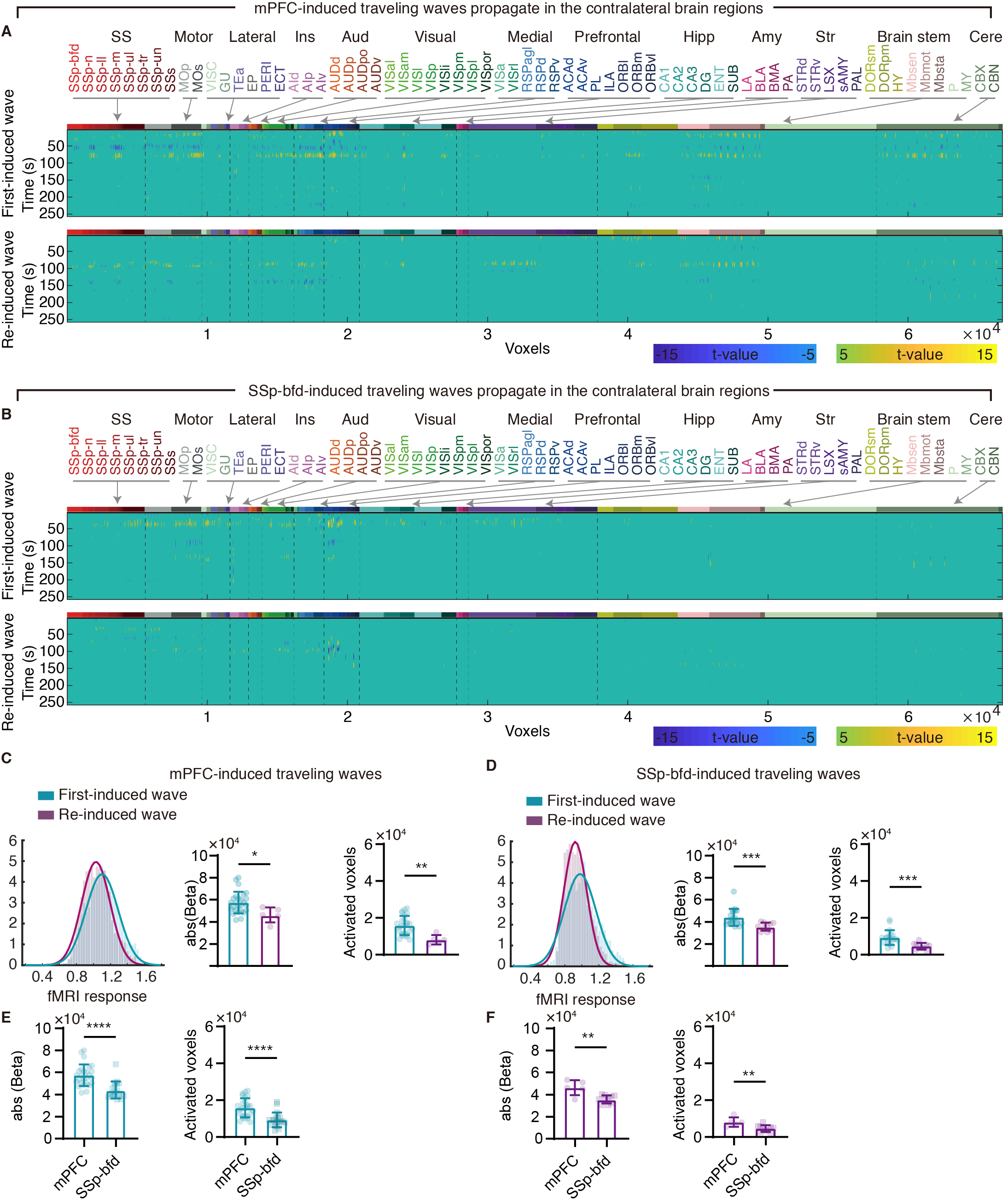


**Figure S8.** Comparison of contralateral propagation properties between mPFC- and SSp-bfd-induced traveling waves. A) Voxel-wise spatiotemporal activation of mPFC first- (Top, a representative mouse) and re-induced traveling waves (bottom, a representative mouse). B) Voxel-wise spatiotemporal activation of SSp-bfd first- (Top, a representative mouse) and re-induced traveling waves (bottom, a representative mouse). C) Quantitative comparisons of the brain responses and activated voxels between mPFC first- and re-induced traveling waves. D) Quantitative comparisons of the brain responses and activated voxels between SSp-bfd first- and re-induced traveling waves. E-F) Quantitative comparisons of the brain responses and activated voxels between mPFC- and SSp-bfd-induced traveling waves. The Mann-Whitney test was used for (D), while two-sample t-tests were applied to all other comparisons. * *P* < 0.05, ** *P* < 0.01. Data are presented as mean±std.


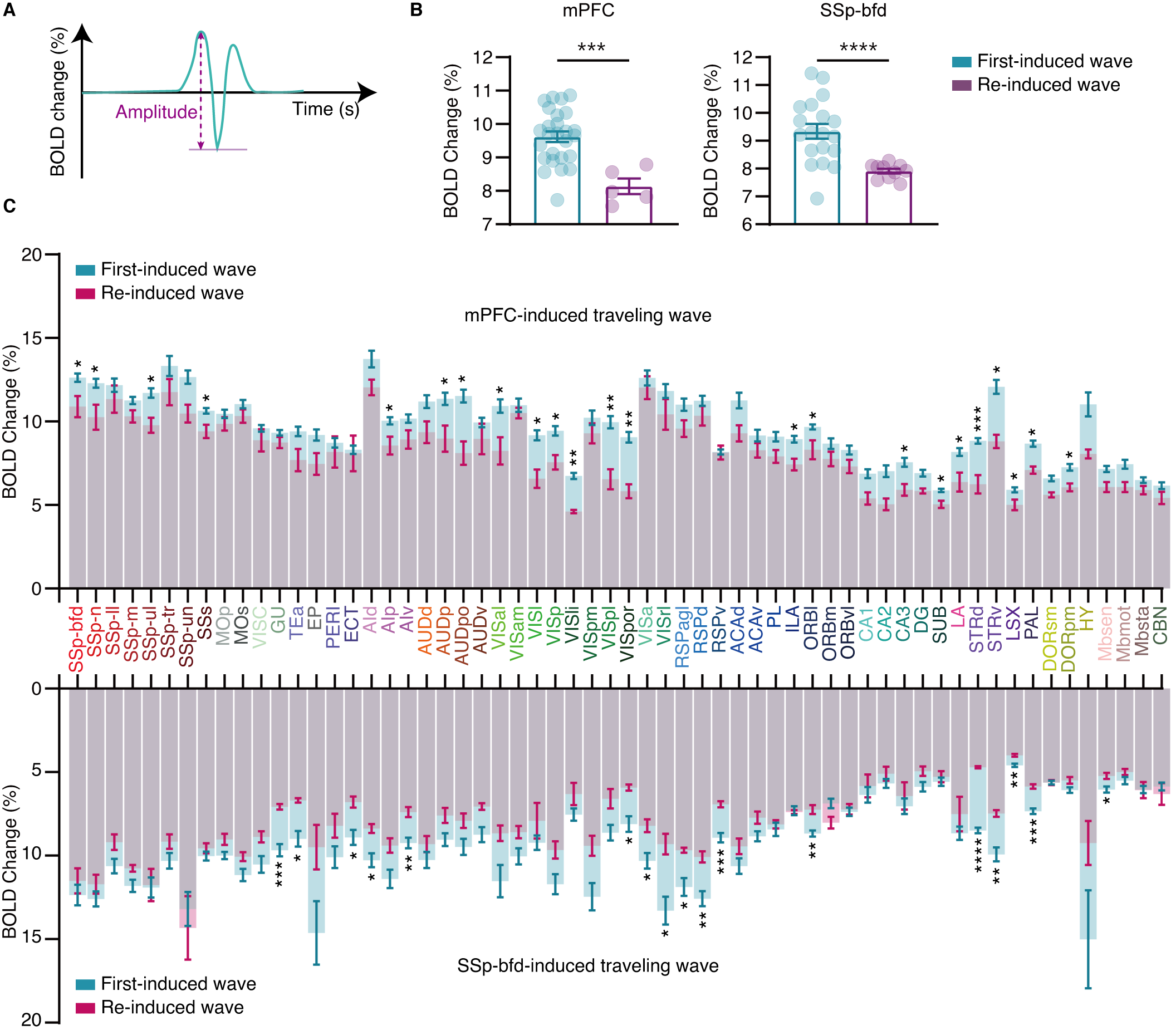


**Figure S9.** BOLD signal amplitude is greater in first-induced traveling waves compared to re-induced traveling waves. A) Definition of BOLD signal amplitude. B) Comparisons of BOLD signal amplitudes averaged over 68 ROIs in first- and re-induced traveling waves induced by optogenetic activation of the mPFC (left, two-sample t-test) and SSp-bfd (right, Mann-Whitney test ). C) Comparisons of BOLD signal amplitude for each of the 68 ROIs in first- and re-induced traveling waves induced by optogenetic activation of the mPFC (top) and SSp-bfd (bottom). * *P* < 0.05, ** *P* < 0.01, *** *P* < 0.001, **** *P* < 0.0001. Data are presented as mean±std.


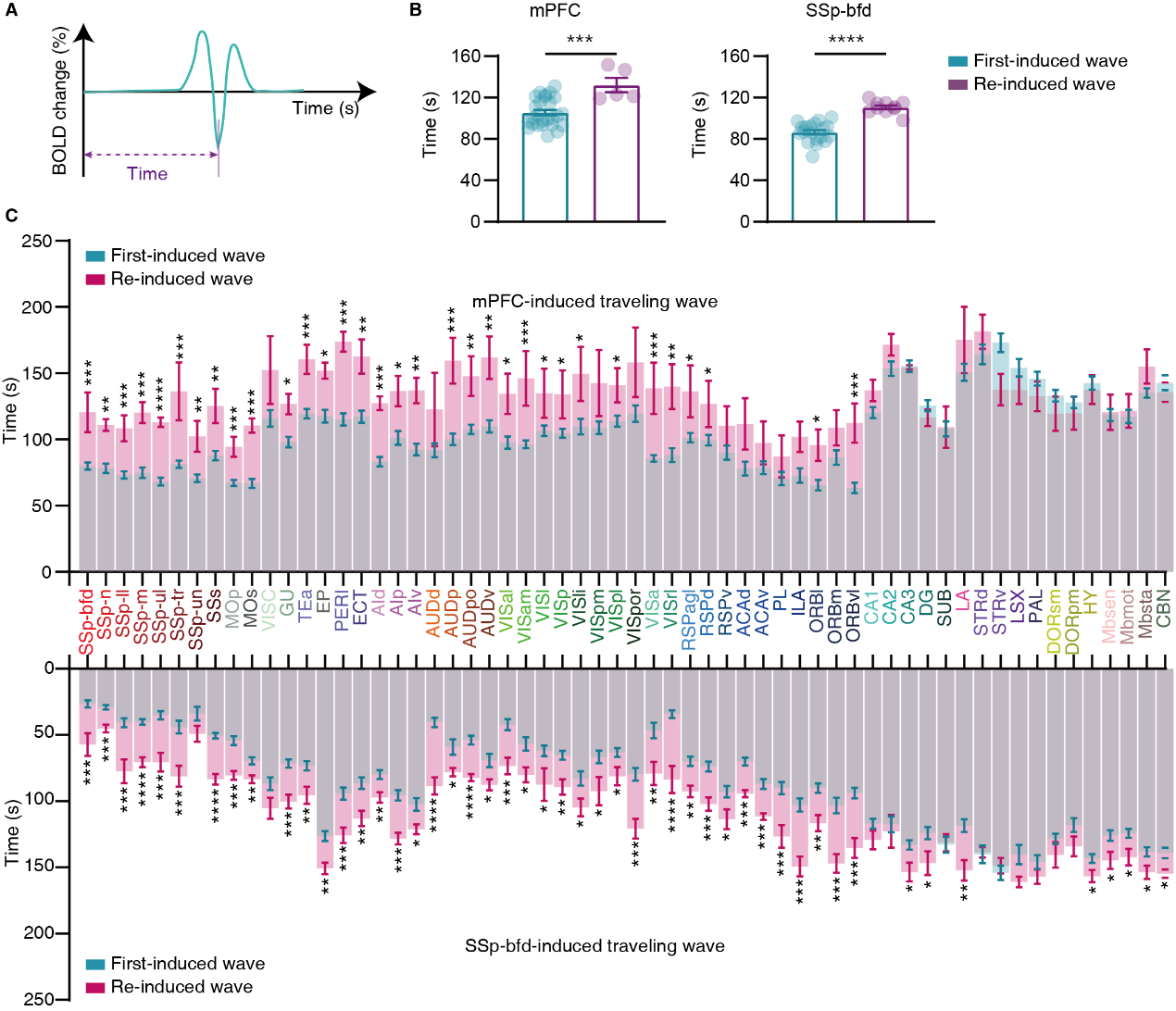


**Figure S10.** First-induced traveling waves propagate faster than re-induced traveling waves. A) The time for CSD-like traveling waves to each ROI was defined as the time corresponding to the negative peak of the BOLD signal. B) Comparisons of propagation time averaged across 68 ROIs in first- and re-induced traveling waves induced by optogenetic activation of the mPFC (left) and SSp-bfd (right). (C) Comparisons of propagation time to each of 68 ROIs in first- and re-induced traveling waves induced by optogenetic activation of the mPFC (top) and SSp-bfd (bottom). Two-sample *t* test, * *P* < 0.05, ** *P* < 0.01, *** *P* < 0.001, **** *P* < 0.0001. Data are presented as mean±std.


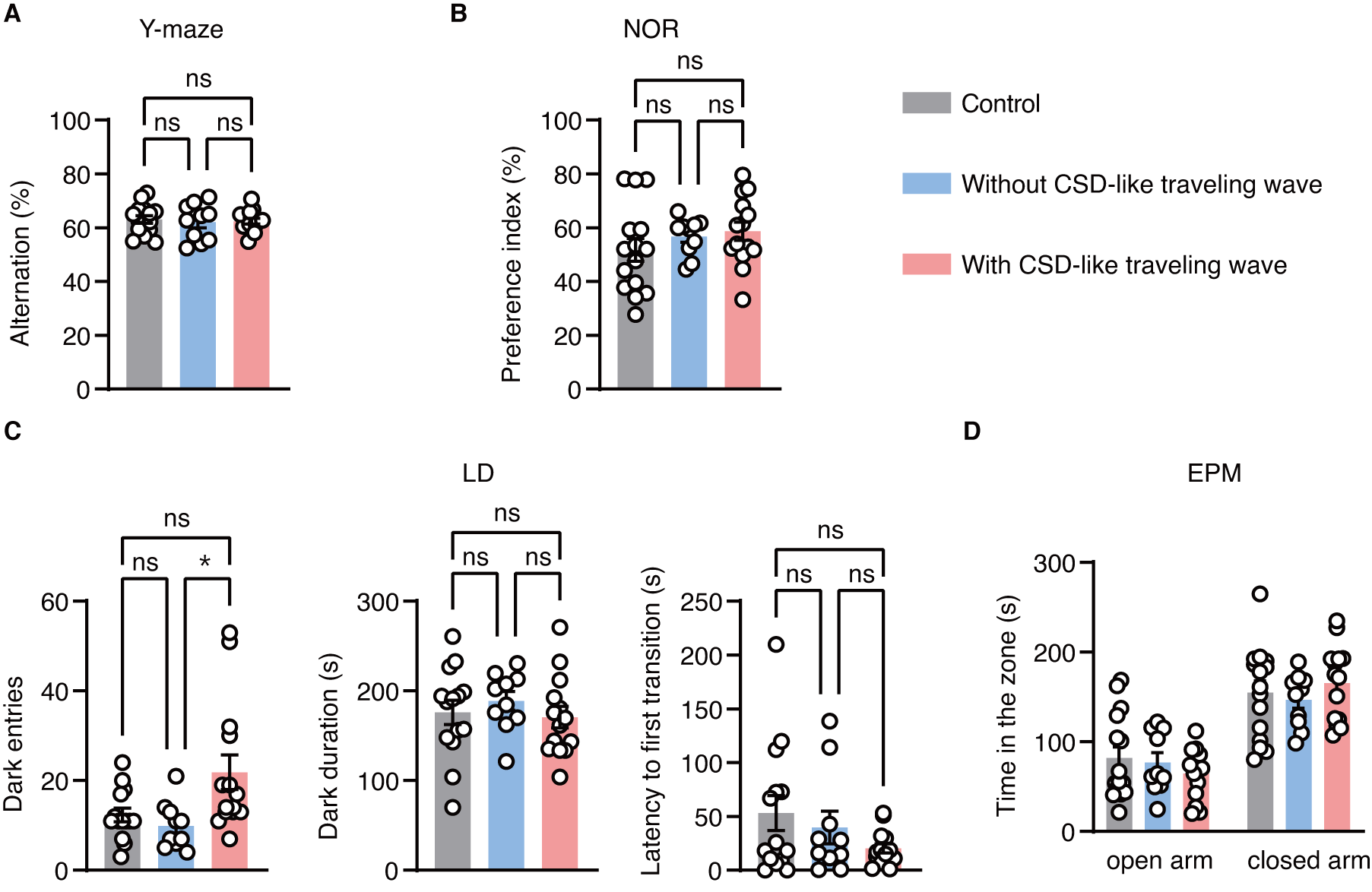


**Figure S11.** Behavioral performances in the Y-maze test (A; *n* = 15, 10 and 14 for control, mice without CSD, and mice with CSD, respectively), novel object recognition test (NOR, B; *n* = 15, 10 and 14 mice), light-dark box test (LD, C; *n* = 14, 10 and 14 mice) and elevated plus maze test (EPM, D; *n* = 15, 10 and 14 mice). One-way ANOVA followed by the Tukey’s multiple comparison test for (A), (D), the dark duration and latency to first transition in the LD test. Kruskal-Wallis test for (B) and the dark entries in the LD test. Data are presented as mean ± s.e.m. ns: no significant difference.

**Table S1.** The definition of 68 ROIs.

| **Brain regions** | **Abbr.** | **Regions of interest (ROIs)** | **Label** | **Brain regions** | **Abbr.** | **Regions of interest (ROIs)** | **Label** |
| --- | --- | --- | --- | --- | --- | --- | --- |
| Somatosensory areas | SSp-bfd | Primary somatosensory area, barrel field | 1 | Prefrontal areas | ACAd | Anterior cingulate area, dorsal part | 37 |
|  | SSp-n | Primary somatosensory area, nose | 2 |  | ACAv | Anterior cingulate area, ventral part | 38 |
|  | SSp-ll | Primary somatosensory area, lower limb | 3 |  | PL | Prelimbic area | 39 |
|  | SSp-m | Primary somatosensory area, mouth | 4 |  | ILA | Infralimbic area | 40 |
|  | SSp-ul | Primary somatosensory area, upper limb | 5 |  | ORBl | Orbital area, lateral part | 41 |
|  | SSp-tr | Primary somatosensory area, trunk | 6 |  | ORBm | Orbital area, medial part | 42 |
|  | SSp-un | Primary somatosensory area, unassigned | 7 |  | ORBvl | Orbital area, ventrolateral part | 43 |
|  | SSs | Supplemental somatosensory area | 8 | Hippocampus | CA1 | Field CA1 | 44 |
| Somatomotor areas | MOp | Primary motor area | 9 |  | CA2 | Field CA2 | 45 |
|  | MOs | Secondary motor area | 10 |  | CA3 | Field CA3 | 46 |
| Lateral areas | VISC | Visceral area | 11 |  | DG | Dentate gyrus | 47 |
|  | GU | Gustatory areas | 12 |  | ENT | Entorhinal area | 48 |
|  | TEa | Temporal association areas | 13 |  | SUB | Subiculum | 49 |
|  | EP | Endopiriform nucleus | 14 | Amygdalar | LA | Lateral amygdalar nuclues | 50 |
|  | PERI | Perirhinal area | 15 |  | BLA | Basolateral amygdalar nucleus | 51 |
|  | ECT | Ectorhinal area | 16 |  | BMA | Basomedial amygdalar nucleus | 52 |
| Agranular insular area | AId | Agranular insular area, dorsal part | 17 |  | PA | Posterior amuygdalar nucleus | 53 |
|  | AIp | Agranular insular area, posterior part | 18 | Striatum | STRd | Striatum dorsal region | 54 |
|  | AIv | Agranular insular area, ventral part | 19 |  | STRv | Striatum ventral region | 55 |
| Auditory areas | AUDd | Dorsal auditory area | 20 |  | LSX | Lateral septal complex | 56 |
|  | AUDp | Primary auditory area | 21 |  | sAMY | Striatum-like amygdalar nuclei | 57 |
|  | AUDpo | Posterior auditory area | 22 |  | PAL | Pallidum | 58 |
|  | AUDv | Ventral auditory area | 23 | Thalamus | DORsm | Thalamus, sensory-motor cortex related | 59 |
| Visual areas | VISal | Anterolateral visual area | 24 |  | DORpm | Thalamus, polymodal association cortex related | 60 |
|  | VISam | Anteromedial visual area | 25 | Hypothalamus | HY | Hypothalamus | 61 |
|  | VISl | Lateral visual area | 26 | Midbrain | Mbsen | Midbrain, sensory-related | 62 |
|  | VISp | Primary visual area | 27 |  | Mbmot | Midbrain, motor-related | 63 |
|  | VISpl | Posterolateral visual area | 28 |  | Mbsta | Midbrain, behavioral state-related | 64 |
|  | VISpm | posteromedial visual area | 29 | Hindbrain | P | Pons | 65 |
|  | VISli | Laterointermediate area | 30 |  | MY | Medulla | 66 |
|  | VISpor | Postrhinal area | 31 | Cerebellum | CBX | Cerebellar cortex | 67 |
| Medial areas | VISa | Anterior area | 32 |  | CBN | Cerebellar nuclei | 68 |
|  | VISrl | Rostrolateral visual area | 33 |  |  |  |  |
|  | RSPagl | Retrosplenial area, lateral agranular part | 34 |  |  |  |  |
|  | RSPd | Retrosplenial area, dorsal part | 35 |  |  |  |  |
|  | RSPv | Retrosplenial area, ventral part | 36 |  |  |  |  |

**Video S1.** The video illustrates the propagation of CSD-like traveling waves as observed directly from the fMRI images.

**Video S2.** The video shows the spatiotemporal dynamic propagation of mPFC-induced cortical spreading depression-like traveling waves throughout the brain.

**Video S3.** The video shows the spatiotemporal dynamic propagation of SSp-bfd-induced cortical spreading depression-like traveling waves throughout the brain.
